# ARG1-expressing microglia show a distinct molecular signature and modulate postnatal development and function of the mouse brain

**DOI:** 10.1038/s41593-023-01326-3

**Published:** 2023-05-11

**Authors:** Vassilis Stratoulias, Rocío Ruiz, Shigeaki Kanatani, Ahmed M. Osman, Lily Keane, Jose A. Armengol, Antonio Rodríguez-Moreno, Adriana-Natalia Murgoci, Irene García-Domínguez, Isabel Alonso-Bellido, Fernando González Ibáñez, Katherine Picard, Guillermo Vázquez-Cabrera, Mercedes Posada-Pérez, Nathalie Vernoux, Dario Tejera, Kathleen Grabert, Mathilde Cheray, Patricia González-Rodríguez, Eva M. Pérez-Villegas, Irene Martínez-Gallego, Alejandro Lastra-Romero, David Brodin, Javier Avila-Cariño, Yang Cao, Mikko Airavaara, Per Uhlén, Michael T. Heneka, Marie-Ève Tremblay, Klas Blomgren, Jose L. Venero, Bertrand Joseph

**Affiliations:** 1 Institute of Environmental Medicine, Toxicology Unit, Karolinska Institutet, Stockholm, Sweden; 2 Neuroscience Center, HiLIFE, University of Helsinki, Helsinki, Finland; 3 Instituto de Biomedicina de Sevilla, IBiS/Hospital Universitario Virgen del Rocío/CSIC, Universidad de Sevilla, Seville, Spain; 4 Department of Medical Biochemistry and Biophysics, Karolinska Institutet, Stockholm, Sweden; 5 Department of Women’s and Children’s Health, Karolinska Institutet, Stockholm, Sweden; 6 Department of Physiology, Anatomy and Cellular Biology, University of Pablo de Olavide, Seville, Spain; 7 Department of Molecular Medicine, Université Laval, and Axe Neurosciences, Centre de Recherche du CHU de Québec-Université Laval, Laval, Quebec Canada; 8 Division of Medical Sciences, University of Victoria, Victoria, British Columbia Canada; 9 Department of Neurodegenerative Diseases and Gerontopsychiatry, University of Bonn, Bonn, Germany; 10 Bioinformatics and Expression Analysis Core Facility, Department of Biosciences and Nutrition, Karolinska Institutet, Stockholm, Sweden; 11 Department of Molecular Biology and Umeå Centre for Microbial Research (UCMR), Umeå University, Umeå, Sweden; 12 Clinical Epidemiology and Biostatistics, School of Medical Sciences, Örebro University, Örebro, Sweden; 13 Unit of Integrative Epidemiology, Institute of Environmental Medicine, Karolinska Institutet, Stockholm, Sweden; 14 Faculty of Pharmacy, Drug Research Program, University of Helsinki, Helsinki, Finland; 15 Luxembourg Centre for Systems Biomedicine, University of Luxembourg, Belvaux, Luxembourg; 16 Department of Infectious Diseases and Immunology, University of Massachusetts Medical School, Worcester, MA USA; 17 Department of Paediatric Oncology, Karolinska University Hospital, Stockholm, Sweden

**Keywords:** Microglia, Development of the nervous system

## Abstract

Molecular diversity of microglia, the resident immune cells in the CNS, is reported. Whether microglial subsets characterized by the expression of specific proteins constitute subtypes with distinct functions has not been fully elucidated. Here we describe a microglial subtype expressing the enzyme arginase-1 (ARG1; that is, ARG1^+^ microglia) that is found predominantly in the basal forebrain and ventral striatum during early postnatal mouse development. ARG1^+^ microglia are enriched in phagocytic inclusions and exhibit a distinct molecular signature, including upregulation of genes such as *Apoe*, *Clec7a*, *Igf1*, *Lgals3* and *Mgl2*, compared to ARG1^–^ microglia. Microglial-specific knockdown of *Arg1* results in deficient cholinergic innervation and impaired dendritic spine maturation in the hippocampus where cholinergic neurons project, which in turn results in impaired long-term potentiation and cognitive behavioral deficiencies in female mice. Our results expand on microglia diversity and provide insights into microglia subtype-specific functions.

## Abstract (web summary)

The molecular diversity of microglia has been described. Here the authors show that ARG1-expressing microglia are enriched in phagocytic inclusions and are involved in hippocampal innervation and spine maturation in mice. ARG1-expressing microglia also modulate cognition in a sex-dependent manner.

## Main

Brain development begins a few weeks after conception and is thought to be complete by early adulthood. Establishment of distinct neural circuits requires the coordination of a complex set of spatial and temporal neurodevelopmental events^1^. In mammals, microglia, the resident immune cells of the CNS, populate the brain from a yolk sac origin during embryogenesis^2–4^ before the overall establishment of neural circuits. This suggests that microglia play roles in brain wiring, supported by microglia-depletion strategies during embryonic stages^5^. Likewise, systemic inflammation during pregnancy affects microglia and exerts deleterious effects on neuronal wiring and contributes to the etiology of neurodevelopmental and neuropsychiatric disorders^6^. Regulation of the composition of the extracellular environment, synaptogenesis, synapse pruning and myelination are all reported microglia-regulated biological processes essential to the emergence of effective neural circuits^7,8^. Roles for microglia are also acknowledged in the context of brain diseases ranging from neurodegenerative disorders, such as Alzheimer’s disease, to neoplasms, including tumors of the developing brain (that is, pediatric tumors)^8–10^. Microglial dysfunction, including the acquisition of neurotoxic or tumor-supporting functions, is a common feature of the aforementioned brain pathologies^9,11–13^. Hence, microglia fulfill multiple functions throughout development and under disease conditions.

Microglia are commonly regarded as a population of versatile cells that can acquire distinct phenotypes after exposure to extrinsic cues in their environment. However, recent high-throughput genome-wide sequencing data revealed that microglia with different transcriptomic profiles coexist throughout the lifespan of mice during both homeostasis and disease-related challenges^14–19^. Whether these subsets constitute different microglial subtypes with intrinsic differences and functional specialization(s) has not been systematically explored^8,9^. Of note, these studies show that microglia heterogeneity is strikingly high during postnatal development when the brain is expanding and establishing its neuronal networks^1^. Postnatal life encompasses critical phases of mammalian brain development. Indeed, whereas the foundation of brain development begins before birth, the wiring of some neuronal networks, in particular those involved in higher cognitive and sensory functions and sex-related behaviors, takes place postnatally. During childhood and adolescence, the brain forms and refines complex neuronal networks through synaptogenesis, pruning and myelination^1^. Interestingly, established microglial biological functions offer a striking match to the above-described postnatal brain developmental events^6^. Furthermore, beyond their immune functions, microglia are reported to modulate the formation of axonal tracks, synaptic reorganization and turnover and activity and contribute to the maturation of neural circuits^5,20,21^. In addition, microglia, in particular CD11c^+^ microglia expressing large amounts of insulin-like growth factor 1, are regulators of oligodendrocyte differentiation and myelin formation^22^. A further emerging dimension of multifaceted microglia is that they exhibit sex differences in morphology, maturation and functional output^23,24^, at least from postnatal development onward. Considering the plethora of functions described for microglia in the developing brain and the reported postnatal microglial transcriptional diversity, one could envisage that distinct microglial subtypes are responsible for exerting these various biological functions.

Here, we report a microglial subtype, arginase-1-expressing (ARG1^+^) microglia. ARG1^+^ microglia are morphologically indistinguishable from neighbouring ARG1^–^ microglia but can be defined by a distinct transcriptomic profile and a unique spatial and temporal distribution and exert a unique function in the developing brain.

## Results

### ARG1^+^ microglia are primarily found in the basal forebrain (BF)

**Fig. 1:**
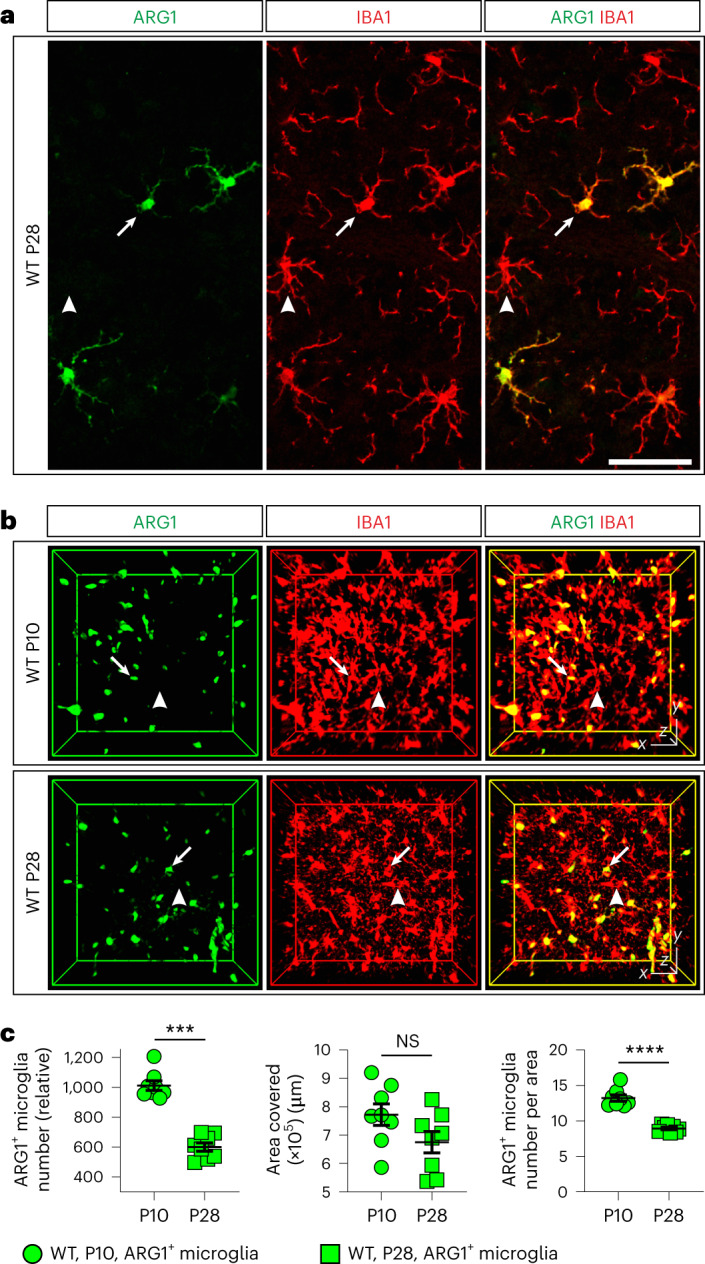
ARG1^+^ microglia coexist in the same vicinity as ARG1^–^ microglia in the BF of P10 and P28 female and male mice. **a**,**b**, ARG1^+^ microglia (arrows) and ARG1^–^ microglia (arrowheads) in WT female (**a**; confocal) and WT male (**b**; iDISCO) mouse brains. Scale bars, *x* = 50 μm and *z* = 8.25 μm (**a**) and *x* = *y* = 50 μm and *z* = 150 μm (**b**). **c**, The ARG1^+^ microglia population declines with age (*n* = 4 female and 4 male animals). Each circle (P10) or square (P28) corresponds to one animal; ***P = 0.0002, ARG1^+^ microglia number (relative); ****P < 0.0001, ARG1^+^ microglia number per area; NS, not significant. Data are shown as mean ± s.e.m. Statistically significant differences were determined by unpaired two-sided *t*-tests (for area covered and ARG1^+^ microglia number per area) and two-sided Mann–Whitney *U*-test (for ARG1^+^ microglia number). Source data

**Extended Data Fig. 1:**
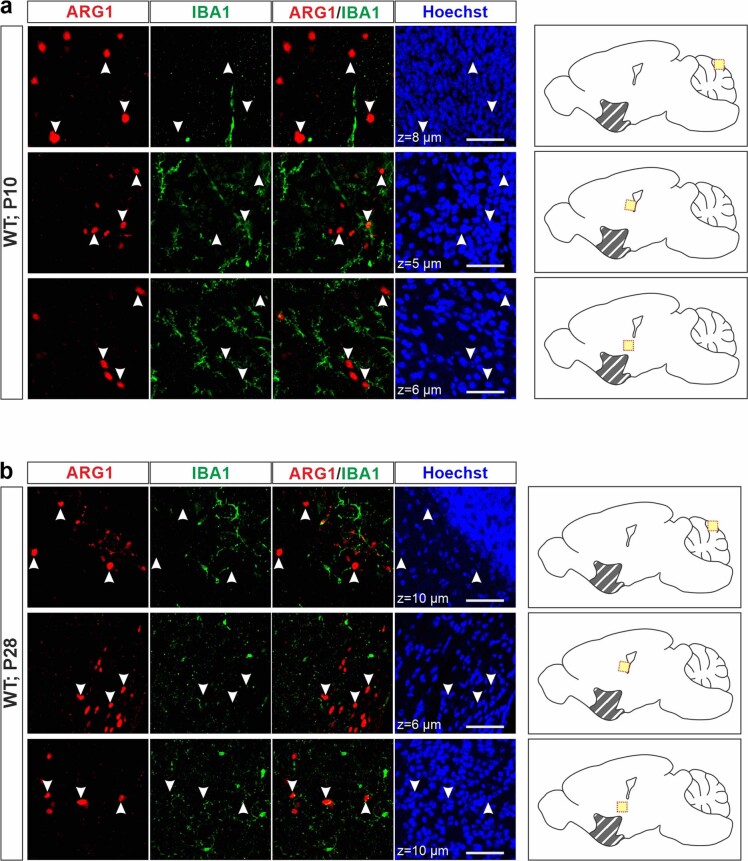
ARG1-positive cells that are not microglia in WT mouse brain. **a-b**, ARG1^+^/IBA1^−^cells in the cerebellum and around the ventricles (arrowheads) both at P10 (**a**) and at P28 (**b**) (representative images from 3 female animals per group). Scale bars, x = 50 μm. Yellow squares indicate location of the corresponding images on their left; grey lines indicate the BF/vStr region.

**Extended Data Fig. 2:**
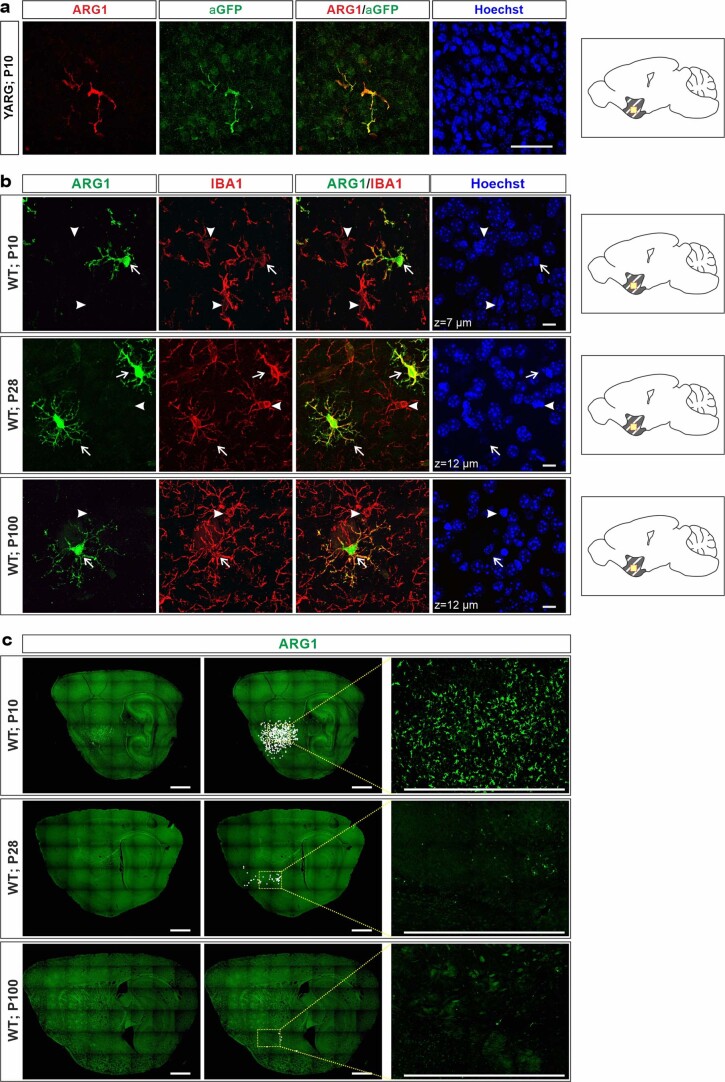
Arg1+microglia co-exist with Arg1^−^negative-microglia in the same vicinity in P10, P28 and P100 WT mouse brain. **a**, *Arg1*-YFP-positive microglia in male YARG mice, as recognised by α-GFP and α-ARG1 antibodies (representative image from 3 female animals). **b**, Arg1+microglia (arrows) and Arg1-negative-microglia (arrowheads) during mouse brain development. **c**, Arg1+microglia in BF/vStr at P10, P28 and P100 (representative images from 3 female animals per group). Each white dot represents a single Arg1+microglia and has been manually annotated. Scale bars, x = 50 μm, z = 10 μm (**a**), x = 10 μm (**b**), x = 1000 μm, z = 4 μm (**c**). Yellow squares indicate location of the corresponding images on their left; grey lines indicate the BF/vStr region.

Using an antibody screen, we identified that in wild-type (WT) and unchallenged brains of mice of both sexes, a subset of microglia coexpresses ionized calcium-binding adaptor molecule 1 (IBA1; encoded by the gene *Aif1*) and the enzyme ARG1. Immunofluorescence analysis of WT mouse brains at postnatal day 10 (P10) and P28 revealed that ARG1^+^ microglia coexist along with ARG1^–^ microglia that do not express ARG1 (Fig. 1a,b). We also identified ARG1^+^IBA1^–^ cells in the cerebellum and around the lateral ventricles, which morphologically do not resemble microglia and were therefore excluded from this study (Extended Data Fig. 1). We further confirmed the existence of ARG1^+^ microglia in YARG reporter mice^25^, which express yellow fluorescent fusion protein (YFP) inserted downstream of the endogenous stop codon of the *Arg1* gene (Extended Data Fig. 2a).

**Fig. 2:**
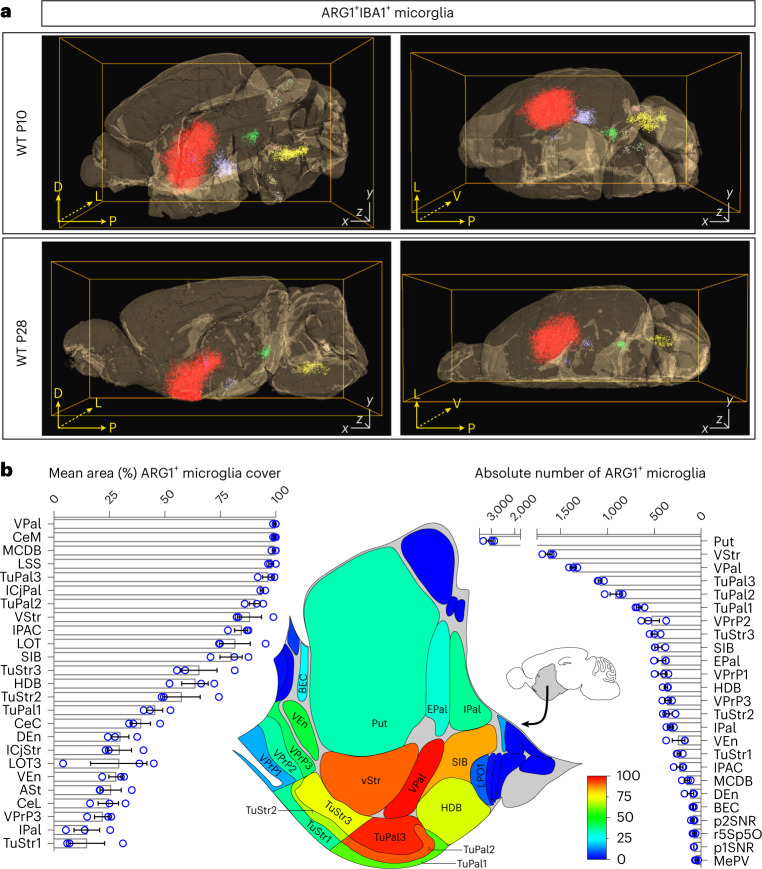
ARG1^+^ microglia have a site-specific distribution in the brains of P10 and P28 WT mice. **a**, ARG1^+^ microglia are found in defined locations in the brain, forming clusters. The largest cluster is found in the BF/vStr (red cluster). Each dot corresponds to a single ARG1^+^IBA1^+^ cell (*n* = 1 animal). Scale bars, *x* = *y* = 1,000 μm and *z* = 5,580 μm (**a**; P10) and *z* = 5,845 μm (**a**; P28). D, dorsal; V, ventral; P, posterior; L, lateral. **b**, Registration of iDISCO+ to the Allen Mouse Brain map of the BF/striatum (*n* = 3 P28 male animals). The percentage of area (pixels) that ARG1^+^ microglia occupy, accompanied by a graphical illustration and absolute number of ARG1^+^ microglia in each brain area, as quantified by iDISCO+ is shown. Each circle corresponds to one animal. Data are shown as mean ± s.e.m. The schematic in **b** was adapted from the Allen Brain Institute Reference Atlas (http://mouse.brain-map.org). Source Data Fig. 2b contains definitions for the abbreviations used. Source data

To gain further insights into the topographical localization of ARG1^+^ microglia, immunolabeling-enabled three-dimensional imaging of solvent-cleared organs (iDISCO+) three-dimensional (3D) deep imaging of ARG1 and IBA1 expression was performed on P10 and P28 mouse brains^26^. ARG1^+^ microglia were found to cluster in several brain regions both at P10 and P28. The ARG1^+^ microglia located in the BF and ventral striatum (vStr) constituted the largest ARG1^+^ microglia cluster (Fig. 2a and Supplementary Videos 1–4). Further registration of the P28 ARG1^+^ microglia population against the Allen Developing Mouse Brain Atlas (http://mouse.brain-map.org) revealed that the highest concentration of ARG1^+^ microglia is located in the ventral pallidum, followed by adjacent areas (Fig. 2b). The BF is an area rich in cholinergic neurons that project to the hippocampus, a structure engaged in cognition^27^, and loss of BF cholinergic projections and reduction of BF volume are associated with reduced cognitive capability^27^.

### ARG1^+^ microglia are abundant in early development

While ARG1^+^ microglia were present at all investigated ages (P10, P28 and P100), and their numbers varied greatly, ARG1^+^ microglia number in the BF/vStr was notably reduced from P10 to P28 (Fig. 1c), while at P100, only a residual population could be observed (Extended Data Fig. 2b,c). Of note, early postnatal life is a critical period for brain development, during which brain size increases and neuronal spines and networks mature, including cholinergic BF neurons^28^. Collectively, these data support the existence of a subset of microglia in the unchallenged WT mouse brain that expresses ARG1 and exhibits intriguing spatiotemporal overlap with the cholinergic system.

### ARG1^+^ microglia do not exhibit morphological aberrations

**Extended Data Fig. 3:**
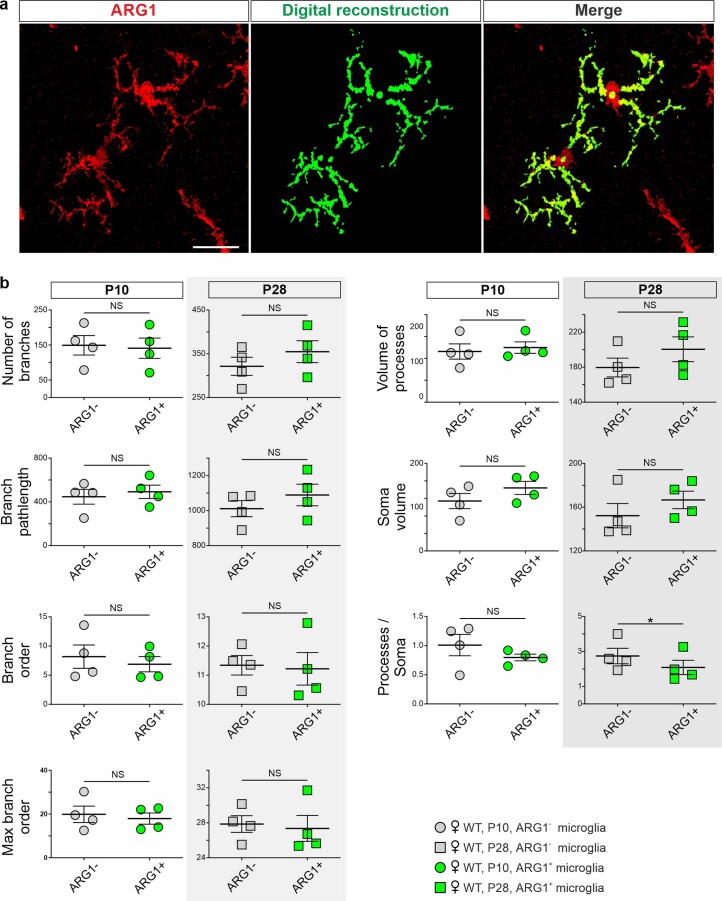
Morphometric comparison of P10 and P28 Arg1-negative-microglia versus Arg1+microglia from basal forebrain. **a**, Illustration of digital reconstruction of two Arg1+microglia. **b**, Morphometric comparisons of Arg1+microglia and Arg1*-*negative-microglia from P10 and P28 mouse brain. Each circle or square represents data from a single animal (n = 4 female animals per group). P28, Processes/Soma, *P* = 0.0259. Scale bar, x = 10 μm, z = 15 μm. Data in **b** represented as mean ± s.e.m. Statistical significances were determined by paired two-sided *t* tests; *P < 0.05, *n.s*. indicates not significant. Source data

We used morphometric analysis to compare ARG1^+^ microglia to neighboring ARG1^–^ microglia from P10 and P28 animals, but no notable morphological differences were observed (Extended Data Fig. 3a,b).

### P13 ARG1^+^ microglia have a unique transcriptomic profile

**Fig. 3:**
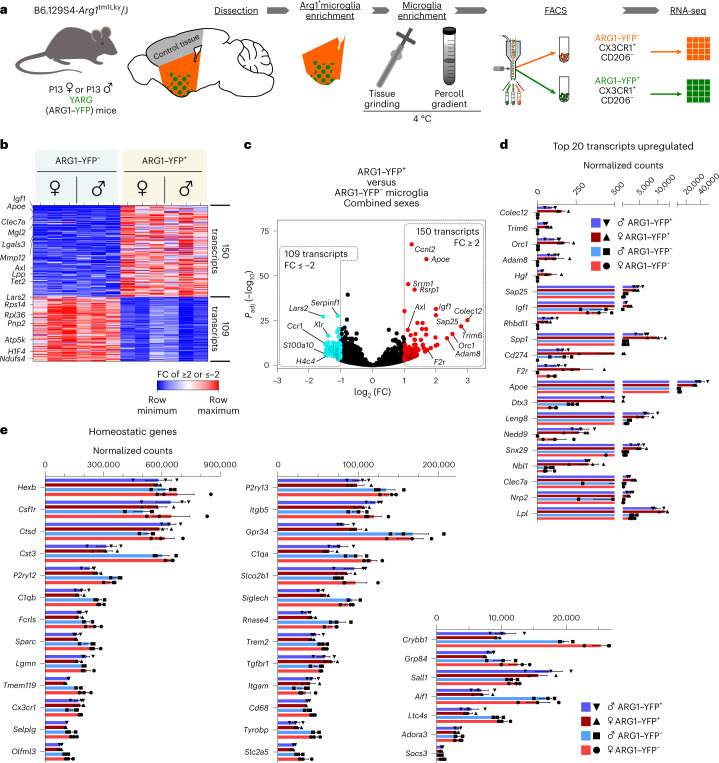
The ARG1^+^ microglia transcriptome is substantially different than that of neighboring ARG1^–^ microglia in female and male P13 mice. **a**, ARG1^+^ microglia were isolated from P13 YARG mice. Three to five brains were dissected from either female or male mice per biological replicate (*n* = 3 litters per sex). Tissues were ground on ice before performing Percoll gradient centrifugation. ARG1^–^ microglia and ARG1^+^ microglia were sorted by flow cytometry, followed by RNA-seq. **b**,**c**, Heat map (**b**) and volcano plot (**c**) of up- and downregulated (at least twofold) genes in ARG1–YFP^+^ and ARG1–YFP^–^ microglia. Only validated genes are included in this list. **d**, List of the 20 most upregulated genes in ARG1–YFP^+^ microglia. **e**, ARG1–YFP^+^ (and ARG1–YFP^–^) microglia express high numbers of transcripts of homeostatic microglial genes. Data in **d** and **e** are shown as mean ± s.e.m. *P* values (two sided) attained by the Wald test are corrected for multiple testing using the Benjamini–Hochberg method (*P*_adj_). Statistically significant differences were not measured for **d** and **e**. Source data

**Extended Data Fig. 4:**
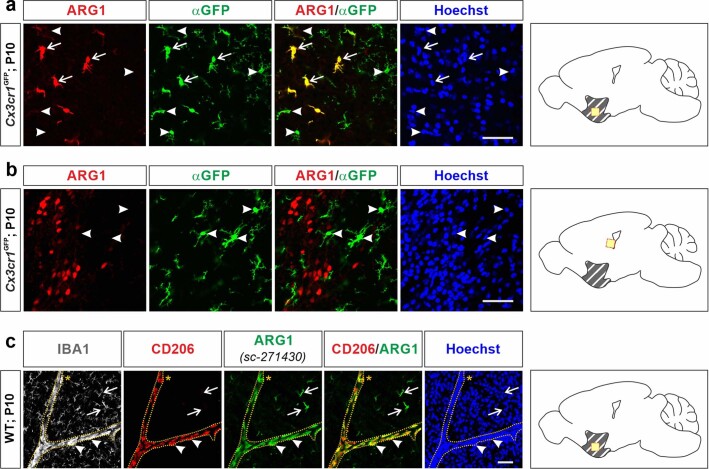
Arg1+microglia co-localize with CX3CR1-GFP but not with CD206 in P10 mouse brains. **a**, CX3CR1-GFP^+^/ARG1 + microglia (arrows) coexist with CX3CR1-GFP^+^/ARG1^−^microglia (arrowheads) in P10 BF/vStr (representative image from 3 female animals). **b**, ARG1-positive cells around the ventricles (triple arrows) are not CX3CR1-GFP-positive (see also Extended Data Fig. 1) (representative image from 3 female animals). **c**, Arg1+microglia do not express the perivascular macrophage marker CD206 (arrows), while perivascular macrophages in this brain area express ARG1 (arrowheads). Note that Arg1+microglia are always ramified, while perivascular macrophages are amoeboid (representative image from 3 female animals). Scale bars, x = 50 μm, z = 10 and 8.5 μm (for **a** and **c**, respectively). Yellow squares indicate location of the corresponding images on their left; grey lines indicate the BF/vStr region.

**Extended Data Fig. 5:**
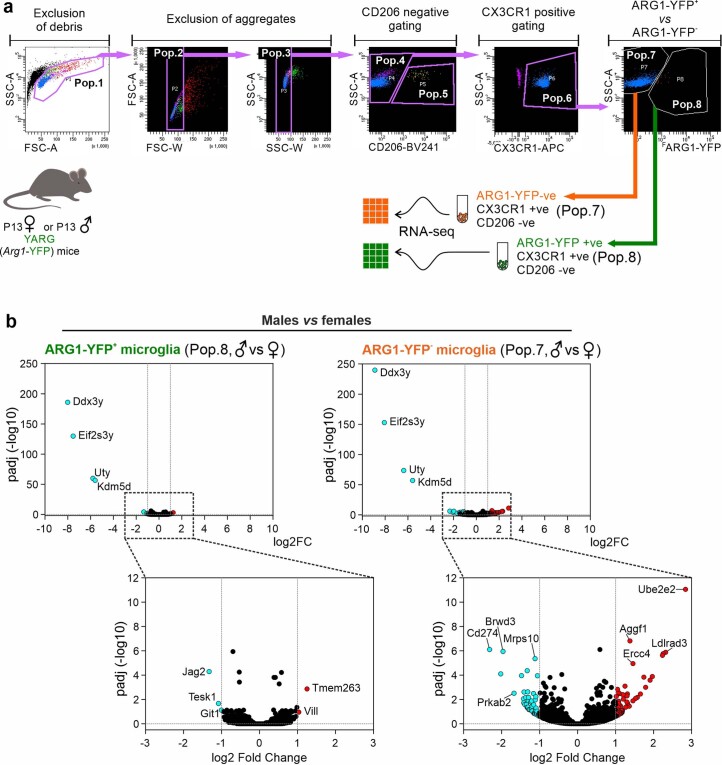
P13 Arg1+microglia from basal forebrain do not have sex-specific signatures. **a**, Gating strategy for fluorescent-activated cell sorting of ARG1*-*YFP-positive (population, Pop.8) and ARG1*-*YFP-negative (Pop.7) microglia. **b**, Volcano plots of differentially expressed genes between males and females in ARG1*-*YFP-positive (Pop.8) and ARG1*-*YFP-negative (Pop.7) sorted microglia. Three to five brains were dissected from either female or male animals per biological replicate (n = 3 litters per sex). P-values (two-sided) attained by the Wald test are corrected for multiple testing using the Benjamini and Hochberg method.

To assess whether ARG1^+^ microglia are characterized by a distinct gene expression profile, we performed bulk RNA-sequencing (RNA-seq) analysis. We isolated ARG1–YFP^+^CX3CR1^+^ microglia and ARG1–YFP^–^CX3CR1^+^ microglia from the area ventral to the corpus callosum and anterior to the lateral ventricles and excluding the olfactory bulb of P13 female and male YARG animals (Fig. 3a). Confirmatory immunohistochemistry analysis in YARG mice showed that ARG1 and YFP protein expression in cells are concurrent (Extended Data Fig. 2a). Furthermore, we validated in CX3CR1–green fluorescent protein (GFP) mice, which express GFP under the control of the endogenous *Cx3cr1* locus, that a subset of CX3CR1-expressing microglia expresses ARG1 in the BF (Extended Data Fig. 4a), while in ventricles, ARG1^+^ cells that morphologically do not resemble microglia do not express CX3CR1–GFP (Extended Data Fig. 4b). We also observed in WT brain vessels ARG1^+^IBA1^+^ cells with amoeboid morphology, which were strongly reminiscent of perivascular macrophages^29^ (Extended Data Fig. 4c). In fact, those cells were positive for the macrophage mannose receptor (CD206)^29^, in contrast to the microglia that were negative for this marker and exhibited a ramified morphology. Based on the above observations, the cell populations of interest were collected by fluorescence-activated cell sorting (FACS) using negative CD206 selection (to exclude perivascular macrophages), positive selection for CX3CR1 (that is, microglial/myeloid marker) and ARG1–YFP expression (Fig. 3a and Extended Data Fig. 5a).

**Extended Data Fig. 6:**
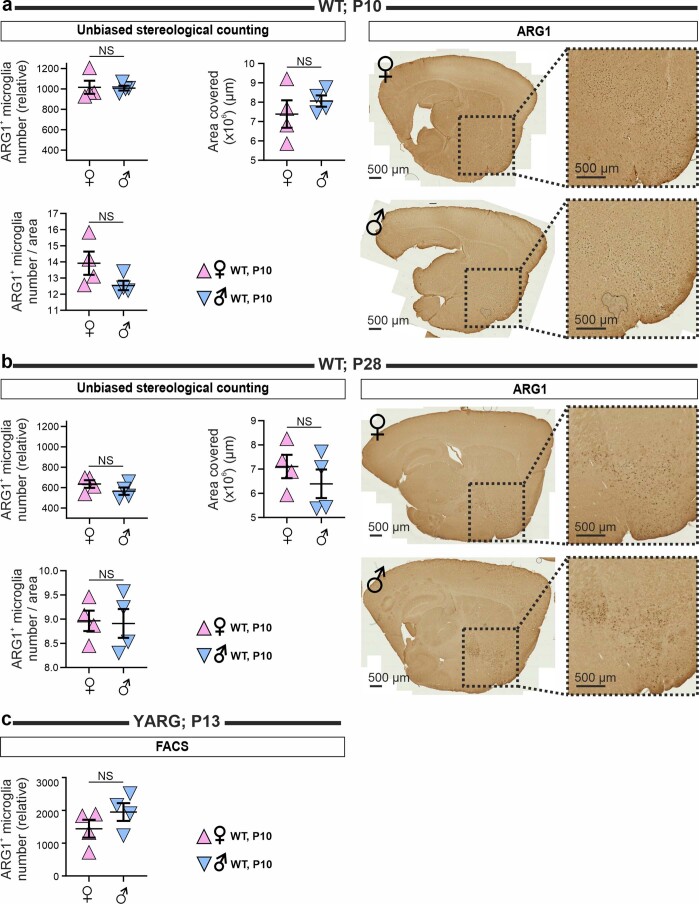
P10, P13 and P28 female and male mice do not have notable differences in Arg1+microglia numbers. **a-b**, Quantification of Arg1+microglia from matching sections of P10 (**a**) and P28 (**b**) wild type animals (n = 4 animals per sex) and representative DAB stainings. **c**, Quantification of ARG1-YFP-positive microglia (Pop.8, as described in Extended Data Fig. 5a) from P13 *Arg1*-YFP female and male animals (n = 4 animals per sex). Each arrow corresponds to one animal. Scale bars, 500 μm. Data represented as mean ± s.e.m. Statistical significances were determined by two-sided unpaired *t* tests (**a**, **b**, and **c)** or two-sided Mann-Whitney U test (for Arg1+microglia number in **a**). *n.s*. indicates not significant. Source data

**Extended Data Fig. 7:**
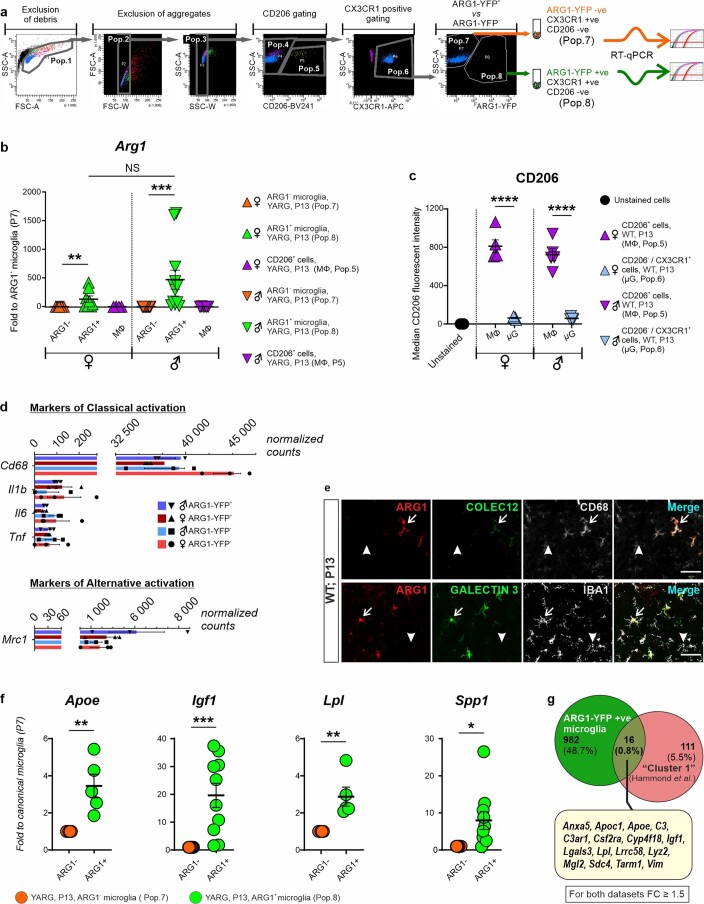
P13 Arg1+microglia cannot be classified as either classical or alternative activated microglia. **a**, Gating strategy for RT-qPCR was identical to gating for sorting prior to RNA-Seq (Extended Data Fig. 5a). **b**, RT-qPCR indicates that *Arg1* gene expression is restricted to the ARG1-YFP + /CX3CR1-/CD206^−^ (Pop.8) population. Three to five brains were dissected from either female or male animals per biological replicate (females, n = 8 litters; males, n = 12 litters). Female, ARG1^negative^ - ARG1^positive^, *P* = 0.0096; male, ARG1^negative^ - ARG1^positive^, *P* < 0.0001. **c**, Microglia (μG) are known to express low levels of *Mrc1* (gene expressing CD206) (**a**, reference^84^), in substantially lower levels than macrophages (MΦ) (females, n = 6 litters; males, n = 5 litters). Female, macrophages - microglia, *P* < 0.0001; male, macrophages - microglia, *P* < 0.0001. **d**, Although ARG1 is long been considered a marker of alternative activation, P13 Arg1+microglia (and P13 Arg1-negative-microglia), express both classical and alternative activation markers (females, n = 3 litters; males, n = 3 litters, data derived from RNA-Seq). **e-f**, Differentially expressed genes from RNA-Seq validated by immunohistochemistry (representative image from 3 female animals) (**e**) and RT-qPCR (n= min. 5 litters) (**f**). *Apoe*, *P* = 0.0049; *Igf1*, *P* = 0.0005; *Lpl*, *P* = 0.0062; *Spp1*, *P* = 0.0101. Scale bars, 50 μm. **g**, Venn diagram showing overlaps between Arg1+microglia and Arg1-negative-microglia presented here and “cluster 1” (reference^17^) (FC ≥ 1.5). Note: only validated genes have been included in this list. Data in **b, c** and **f** are represented as mean ± s.e.m. Statistical significances were determined by Kruskal-Wallis test (**b**, Arg1), ANOVA (**c**, CD206), and unpaired *t* tests (**f**); all P-values are two-sided, and P-values for multiple comparisons were corrected using Dunn’s method (**b**) and Bonferroni’s method (**c**), respectively. *P < 0.05; **P < 0.01; ***P < 0.001; ****P < 0.0001, *n.s*. indicates not significant. Source data

Three independent biological replicates from pooled female or male mouse brain tissues were used for transcriptomic analysis. RNA-seq data revealed that ARG1^+^ microglia possess a unique and distinct transcriptomic profile compared to ARG1^–^ microglia from the same brain area. One hundred and fifty genes were upregulated and 109 genes were downregulated at least twofold in ARG1^+^ microglia compared to in ARG1^–^ microglia (Fig. 3b,c and Supplementary Table 1). The transcriptomes of ARG1^+^ microglia from P13 females and males were almost indistinguishable and showed substantially less sex-dependent variation than ARG1^–^ microglia (Extended Data Fig. 5b). Also, the numbers of ARG1^+^ microglia in males and females were similar, as shown by unbiased stereological counting and FACS (Extended Data Fig. 6). Despite the detection of substantial microglial ARG1 (as well as YFP for YARG mice) protein expression even at P28 in the BF of unchallenged WT and YARG mice, *Arg1* transcripts were not observed in the RNA-seq analysis for P13 ARG1–YFP^+^CX3CR1^+^CD206^−^ microglia (Supplementary Table 1). However, quantitative PCR with reverse transcription (RT–qPCR) analysis performed on sorted ARG1–YFP^+^CX3CR1^+^CD206^–^ and ARG1–YFP^−^CX3CR1^+^CD206^−^ cell populations using the same extraction pipeline confirmed restriction of the expression of *Arg1* gene expression to the ARG1^−^YFP^+^CX3CR1^−^CD206^−^ population (Extended Data Fig. 7a–c). ARG1^+^ microglia express high mRNA copy numbers of microglial homeostatic genes, such as *P2ry12*, *Tmem119*, *Siglech*, *Gpr34*, *Socs3*, *Hexb*, *Olfml3* and *Fcrls*^30^, confirming that these cells are indeed microglia (Fig. 3e). Of note, most of these microglial homeostatic genes are expressed at lower levels in ARG^+^ than in ARG1^–^ microglia from the same brain area, a feature that has been reported for reactive^31^ and disease-associated microglia^32,33^.

**Extended Data Fig. 8:**
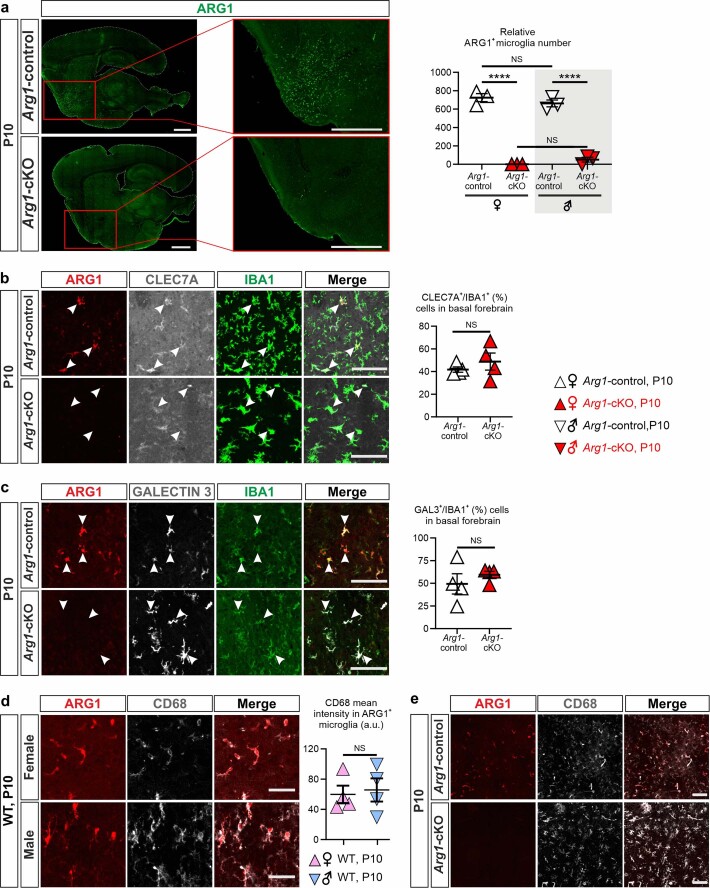
Arg1+microglia are efficiently knocked down in female and male Arg1-cKO. **a**, Staining of matching sections shows that in *Arg1-cKO* animals, only few Arg1+microglia remain when compared to *Arg1-Control*. Quantification of Arg1+microglia from matching sections (n = 3 animals per group). Female, *Arg1*^*Control*^ – *Arg1*^*cKO*^, *P* < 0.0001; male, *Arg1*^*Control*^ – *Arg1*^*cKO*^, *P* < 0.0001. Scale bar, x = 1000 μm. **b**, Arg1+microglia co-localize with the marker GALECTIN-3, as inferred by our RNA-Seq analysis. Scale bar, x = 100 μm, z = 10 μm. In *Arg1-cKO* basal forebrain, the percentage of CLEC7A +/IBA1 + cells is not statistically significant different to the percentage of CLEC7A + /IBA1 + cells in the basal forebrain of *Arg1-Control* animals. **c**, Similar for GALECTIN-3 + /IBA1 + cells. **d**, Mean intensity of the lysosomal marker CD68 in female and male Arg1+microglia in BF. The images in **b, c** and **e** are representative of 4 female animals per group. Scale bars, x = 50 μm, z = 5 μm. **e**, CD68 expression in BF of *Arg1-Control* and *Arg1-cKO* basal forebrain. Scale bars, x = 100 μm, z = 5 μm. Data in **a-d** are represented as mean ± s.e.m. Statistically significant differences were determined by ANOVA (**a**), Mann-Whitney U (**b**), or unpaired *t* tests (**c** and **d**). All P-values are two-sided, and P-values for multiple comparisons were corrected using Bonferroni’s method (a). ****P < 0.0001, *n.s*. indicates not significant. Source data

Although *Arg1* gene expression has been traditionally associated with the outdated term alternative microglia, our RNA-seq analysis showed that ARG^+^ microglia cannot be classified as such (Extended Data Fig. 7d). Instead, ARG^+^ microglia are characterized by high expression of genes such as *Axl*, *Apoe*, *Clec7a*, *Mgl2*, *Lgals3 and Igf1* (ref. ^30^; Fig. 3b–d and Supplementary Table 1). Coexpression of GALECTIN-3 (encoded by the gene *Lgals3*), COLEC12 and CLEC7A proteins and *Apoe*, *Igf1*, *Lpl* and *Spp1* transcripts in ARG1^+^ microglia was further validated by immunohistochemistry and qPCR, respectively (Extended Data Figs. 7e,f and 8b,c). A recent high-throughput microglia single-cell transcriptomic analysis revealed several distinct microglial subsets^17^. In the context of the current investigation, Hammond et al.^17^ found that a particular subtype of microglial cells, defined as cluster 1, showed elevated levels of *Arg1* expression at young ages. Direct comparison of the ARG1^+^ microglia from this study and cluster 1 genes^17^ revealed that both microglial types share a transcriptomic signature of 16 genes (with a fold change (FC) of >1.5), including upregulation of *Apoe*, *C3*, *Lgals3*, *Mgl2* and *Igf1* (Extended Data Fig. 7g).

### ARG1^+^ microglia have increased numbers of cellular inclusions

**Fig. 4:**
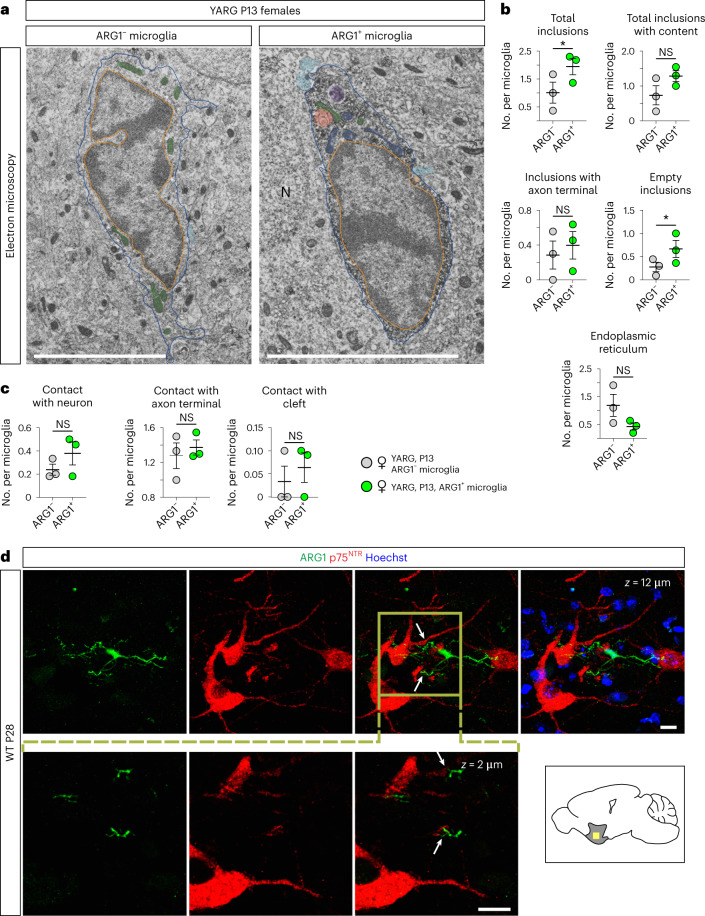
ARG1^+^ microglia contain more inclusions than ARG1^–^ microglia in the BF of P13 YARG mice. **a**, Representative transmission electron microscopy images showing the ultrastructure of ARG1^–^ and ARG1^+^ microglia from P13 YARG female brains in the BF/vStr. **b**,**c**, Quantitative analysis of intracellular features (**b**) and intracellular relationships (**c**; *n* = 3 female animals); total inclusions, *P* = 0.0283; empty inclusions, *P* = 0.0454. **d**, In the WT P28 BF/vStr, ARG1^+^ microglia are in close proximity to cholinergic neurons, as detected with an antibody to p75^NTR^ (*n* = 3). Scale bars, 5 μm. The yellow square indicates the location of the corresponding images below; cyan, axon terminals; red, inclusion with content; orange, empty inclusion; green, mitochondria; blue, holy mitochondria; purple, secondary lysosome; N, neuron; asterisk, endoplasmic reticulum; blue line, cell membrane; orange line, nuclear membrane. Data are shown as mean ± s.e.m. Statistically significant differences were determined by paired two-sided *t*-tests (for total inclusions, total inclusion with content, inclusion with axon terminal, empty inclusion, endoplasmic reticulum, contact with neuron and contact with axon terminal) or two-sided Wilcoxon matched-pairs signed-rank test (for contact with cleft); *P ≤ 0.05. Source data

Ultrastructural analysis of ARG1^+^ microglial cell bodies in the BF/vStr of female mice revealed a higher number of empty phagocytic inclusions and total number of inclusions, which considered empty and inclusions with content together, than in ARG1^–^ microglia, indicating increased phagocytic activity (Fig. 4a–c). Although there was no notable difference in the number of direct contacts between ARG1^+^ microglia and synaptic elements, some of the inclusions contained structures with synaptic vesicles, implying the phagocytosis of presynaptic axon terminals^34^ and involvement in neural development. The proximity of ARG1^+^ microglia to cholinergic neurons was verified by immunohistochemistry (Fig. 4d).

### *Arg1* microglial deletion impacts cognition in female mice

**Fig. 5:**
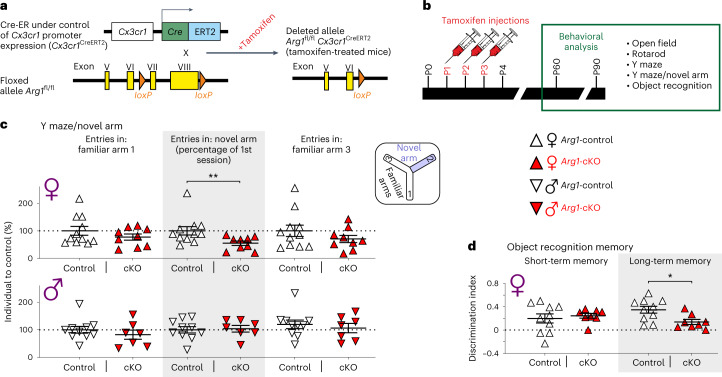
*Arg1* microglial cKO leads to an impaired cognition phenotype in 2- to 3-month-old female mice. **a**,**b**, Strategy for *Arg1*-cKO and subsequent behavioral studies. **c**, Percentage of entries in each arm in relation to the percentage of the first session was determined; entries in novel arm, *P* = 0.0058. **d**, Short-term memory and long-term memory were expressed as a discrimination index (number novel – number familiar)/(number novel + number familiar) taking into account the training index; long-term memory, *P* = 0.0148. Each triangle corresponds to one animal; female *Arg1*-control *n* = 11 (**c**) and *n* = 10 (**d**) and *Arg1*-cKO *n* = 9 (**c**) and *n* = 8 (**d**); male *Arg1*-control *n* = 10 (**c**) and *Arg1*-cKO *n* = 6 (**c**). Data are shown as mean ± s.e.m. Statistically significant differences were determined by two-sided Mann–Whitney *U*-test (for females Y maze/novel arm) and unpaired two-sided *t*-tests (for males Y maze/novel arm and females object recognition memory); *P < 0.05 and **P ≤ 0.01. Source data

Given the unique spatiotemporal distribution and substantially different transcriptomes of ARG1^+^ microglia compared to neighboring ARG1^–^ microglia, we sought to investigate if they also have a distinct functional specialization. We specifically knocked out the *Arg1* gene in microglia by crossing *Cx3cr1*^CreER^ mice with *Arg1*^fl/fl^ mice and induced recombination by multiple tamoxifen injections from P1 (Fig. 5a,b). Using immunohistochemistry, we confirmed that ARG1 expression was suppressed efficiently (Extended Data Fig. 8a). Co-staining for GALECTIN-3 and CLEC7A proteins, which are coexpressed in ARG1^+^ microglia in WT mice, revealed that in *Arg1*-knockout animals, the number of GALECTIN-3- and CLEC7A-expressing microglia persisted in the BF at similar numbers as observed in WT littermates (Extended Data Fig. 8b,c). This implies that after *Arg1* knockout, ARG1^+^ microglia stop expressing ARG1, but they do not cease to exist. Future in vivo lineage-tracing studies would certainly be informative to decipher ARG1^+^ microglia ontogeny and maturation.

**Extended Data Fig. 9:**
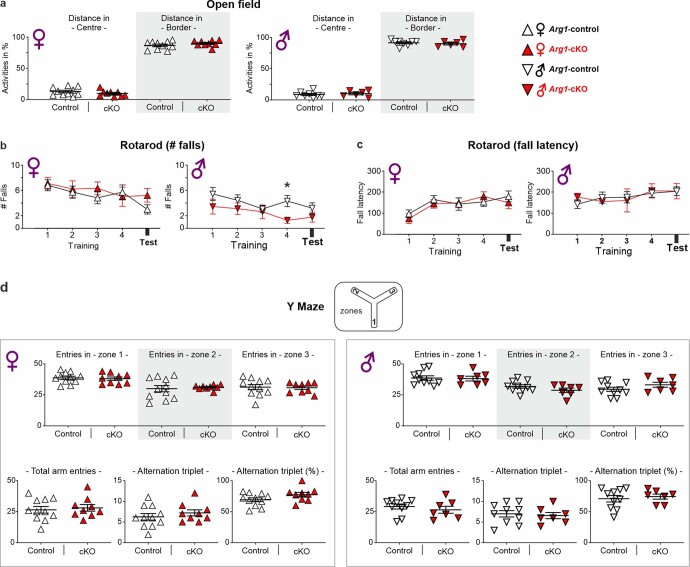
Arg1-cKO female and male animals do not show motoric phenotypes. **a**-**e**, *Arg1-cKO* animals and controls were assessed for motoric (**a**-**c**) and memory (**d**) phenotypes. Each triangle corresponds to one animal. Female *Arg1-Control* n = 10 (**a-c**), n = 11 (**d**) and *Arg1-cKO* n = 9 (**a-d**); male *Arg1-Control* n = 8 (**a-c**), n = 10 (**d**) and *Arg1-cKO* n = 6 (**a-c**), n = 7 (**d**). Males, rotarod #falls, training 4, *P* = 0.0184. Data represented as mean ± s.e.m. Statistically significant differences were determined by unpaired two-sided *t* tests. *P < 0.05. Source data

The topographic organization of ARG1^+^ microglia proximal to the cholinergic nucleus of the BF, a major nucleus for cognition^27^, and the cognitive deficiencies described after *ARG1* impairment in humans^35,36^ prompted us to investigate if cognition was affected in conditional *Arg1*-knockout animals (*Arg1*^fl/fl^; *Cx3cr1*^CreER+/–^; hereafter referred to as *Arg1*-cKO) compared to control animals (*Arg1*^fl/fl^; *Cx3cr1*^CreER−/−^; hereafter referred to as *Arg1*-control). Two- to 3-month-old *Arg1*-cKO female and male mice did not display motor coordination dysfunction compared to *Arg1*-control mice (Extended Data Fig. 9a–c). To study hippocampus-dependent spatial memory, we used the novel arm discrimination (spatial recognition memory) paradigm in the Y maze. This test is based on the inherent preference of mice to explore a novel environment more than a familiar one. The spontaneous alternation triplets percentage was similar in female and male experimental groups (Extended Data Fig. 9d), indicating that working memory seems to be unaffected when *Arg1* is depleted in microglia. Interestingly, *Arg1*-cKO female mice showed a statistically significant decrease in the percentage of times they visited the new arm compared to *Arg1*-control mice (Fig. 5c), meaning that the absence of *Arg1* expression in microglia impaired cognitive function in the mouse model. To gain further insights, we performed an object recognition memory test. *Arg1*-cKO female mice had a reduced preference for a new object compared to a familiar object 24 h after the training session, therefore exhibiting impairment in long-term memory acquisition (Fig. 5d). No differences were found in the short-term memory index (Fig. 5d) measured 1 h after the training protocol, indicating once more that working memory was unaffected in the experimental groups. By contrast, we were not able to detect any behavioral phenotype in *Arg1*-cKO male mice (Fig. 5c). This sex-specific phenotype cannot be attributed to differences in ARG1^+^ microglia transcriptomic profiles (Extended Data Fig. 5b) or numbers of cells between sexes (Extended Data Fig. 6). The analysis of the mean intensity of the lysosomal marker CD68 did not reveal differences between female and male ARG1^+^ microglia (Extended Data Fig. 8d,e). Whether the observed behavior difference between male and female mice after microglial *Arg1* knockout could be linked to the reported regulation of ARG enzymes by steroid hormones^37^ requires further investigation. Yet, similar sex differences have been reported when *Arg1* is depleted in peripheral myeloid cells^38^.

### *Arg1* microglial deletion impacts cholinergic innervation

**Extended Data Fig. 10:**
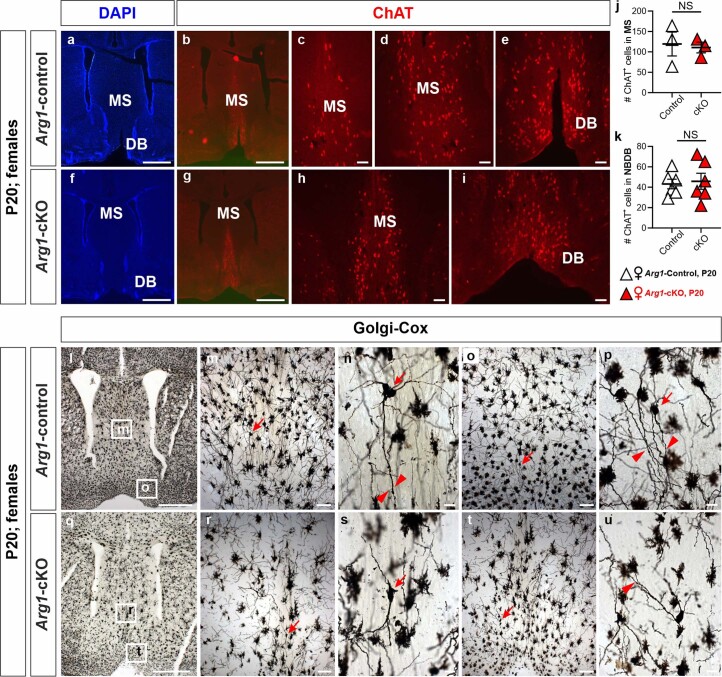
P20 female medial septum and Broca’s diagonal band do not show differences in number of ChAT positive neurons between Arg1-Control and Arg1-cKO brains. **a-i**, Microphotographs of coronal sections of *Arg1-Control* (**a-e** and **l-p**) and *Arg1-cKO* (**f-i** and **q-u**) P20 forebrain. Choline acetyltransferase immunoreactive cells somata are uniformly distributed through the medial septum (MS; **b-d** and **g-h**) and the Broca’s diagonal band (DB; **e** and **i**). **j-k**, ChAT positive neuron cell count in MS and NBDN did not reveal differences between the two genotypes (**j**, n = 3 and **k**, n = 6 animals)**. l-u**, Golgi-Cox method shows the morphology of the neurons of the medial septum (**m-n** and **r-s**) and the diagonal band of Broca (**o-p** and **t-u**). Dendrites with long spines (arrowheads) arose from fusiform or triangular-shaped neuronal bodies (arrows), and not differences between *Arg1-Control* and *Arg1-cKO* neurons are evident. Microphotographs **a** and **f** are DAPI counterstained to help in the identification of anatomical landmarks. Low magnification **l** and **q** microphotographs illustrate the medial septum (squares, **m** and **r)** and the diagonal band of Broca (squares, **o** and **t**) areas. Scale bars, 1000 µm (**a**, **f**, **l** and **q**), 100 µm (**b**, **c**, **g**, **h**, **m**, **o**, **r**, **t**), and 20 µm (**d**, **e**, **h**, **i**, **n**, **p**, **s**, **u**). Data in **j** and **k** are in mean ± s.e.m. Statistical significances were determined by unpaired two-sided *t* tests. *n.s*. indicates not significant. Source data

The forebrain cholinergic system has been involved in the maintenance of hippocampal neurons and in learning, memory and other behavioral processes^39^. Cholinergic inputs to the hippocampus arise from the medial septal (MS) nucleus and the nucleus of the diagonal band of Broca (DB^40^; areas adjacent to ARG1^+^ microglia localization), whose cholinergic fibers distribute throughout the molecular layer of the dentate gyrus (DG) and the stratum oriens of CA1 to CA3 hippocampal areas^40,41^. Therefore, we focused our analysis on these cholinergic regions. Choline acetyltransferase (ChAT)-immunoreactive cell somata were uniformly distributed through the areas of both the MS and DB nuclei in female *Arg1*-control and *Arg1*-cKO brains, with no evident qualitative differences (Extended Data Fig. 10a–i). Stereological analysis also did not reveal differences in the number of ChAT^+^ cells in either nuclei between *Arg1*-control and *Arg1*-cKO forebrains (Extended Data Fig. 10j,k). Golgi–Cox preparations showed that MS and DB neurons possess a triangular or fusiform cell body from which arises several dendrites that, preferentially, run vertically^42^ (Extended Data Fig. 10l–u). Long and thin spines are present through the lengths of the dendritic branches, and no qualitative differences were observed in spine length and/or number between *Arg1*-control and *Arg1*-cKO P20 mice (Extended Data Fig. 10).

**Fig. 6:**
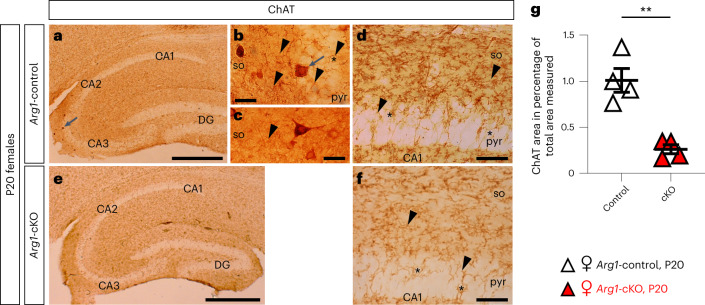
The *Arg1*-cKO hippocampi of 2- to 3-month-old female mice receive reduced cholinergic innervation. **a**–**f**, Microphotographs of sagittal sections of *Arg1*-control (**a**–**d**) and *Arg1*-cKO (**e**,**f**) P20 female hippocampi. ChAT immunoreactivity was revealed by using DAB as a chromogen. Triangular or ovoid immunoreactive ChAT interneurons were observed in the CA3 field of the *Arg1*-control hippocampus (**a**,**b**, arrows). Cholinergic axons show the characteristic varicosities (**b**–**d**,**f**, arrowheads) of the boutons of en passant synapses. At the pyramidal cell layer (pyr), immunoreactive fibers delineate the pyramidal neuronal somata (so; **d**,**f**, asterisk). **g**, Quantitative analysis demonstrates that the female *Arg1*-cKO hippocampus receives less cholinergic innervation than the *Arg1*-control hippocampus; *P* = 0.0016. Each triangle corresponds to one animal (*n* = 4). Scale bars, 500 µm (**a**,**e**), 50 µm (**d**,**f**) and 20 µm (**b**,**c**). Data are shown as mean ± s.e.m. Statistically significant differences were determined by an unpaired two-sided *t*-test; **P ≤ 0.01. Source data

Immunohistochemistry against ChAT revealed cholinergic fibers distributed through the entire hippocampus (Fig. 6a–f), which were more concentrated in the molecular layer of the DG and in the stratum oriens of CA1. Interneuron ChAT^+^ cell somata have been described in the rodent hippocampus^41^. In *Arg1*-control hippocampi, ChAT^+^ interneurons were often observed throughout the stratum oriens and its border with the pyramidal cell layer of CA3 (Fig. 6a,b), while they were absent in the *Arg1*-cKO hippocampus (Fig. 6e). In addition, we identified differences in the density of ChAT^+^ fibers that innervate the hippocampi of the two different genotypes. Although they display the same morphological features as axonal varicosities indicative of en passant synapses and the delineation of pyramidal cell bodies (Fig. 6d,f), fewer ChAT^+^ fibers innervate the *Arg1*-cKO hippocampus (Fig. 6g).

### *Arg1* microglial deletion impacts spine maturation

**Fig. 7:**
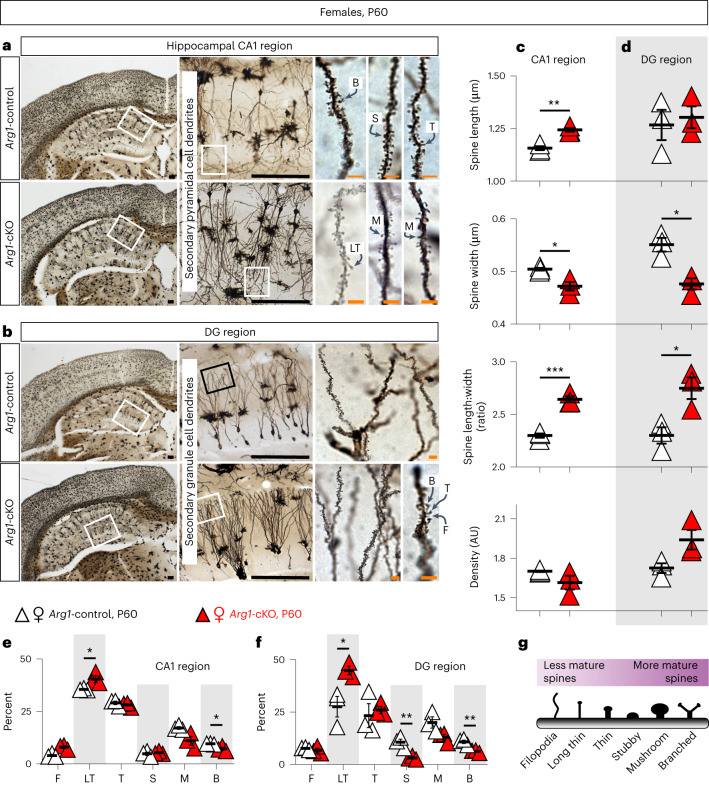
*Arg1* microglial cKO affects dendrite maturation in the hippocampus of 2- to 3-month-old female mice. **a**,**b**, Coronal Golgi–Cox-stained sections of female P60 *Arg1*-cKO and control brains. Hippocampal CA1 and DG regions in which secondary pyramidal and secondary granule cell dendrites arise from the main one, respectively, were used for spine counts. **c**–**f**, Graphical representation of differences between spines in the hippocampal CA1 (**c**,**e**) and DG (**d**,**f**). CA1 length, *P* = 0.0023; CA1 width, *P* = 0.0191; CA1 length:width, *P* = 0.0006; DG width, *P* = 0.0114; DG length:width, *P* = 0.0251; CA1 long thin (%), *P* = 0.0571; CA1 branched (%), *P* = 0.0273; DG long thin (%), *P* = 0.0318; DG stubby (%), *P* = 0.0087; DG branched (%), *P* = 0.0066; AU, arbitrary units. **g**, The six main spine categories according to their morphological characteristics, including filopodia (F), long thin (LT), thin (T), stubby (S), mushroom (M) and branched (B). Each triangle corresponds to one animal (female *Arg1*-control *n* = 3; female *Arg1*-cKO *n* = 3; male *Arg1*-control *n* = 2; male *Arg1*-cKO *n* = 3). Black scale bars, 200 μm. Orange scale bars, 5 μm. Data are shown as mean ± s.e.m. Statistically significant differences were determined by unpaired two-sided *t*-tests or two-sided Mann–Whitney *U*-test (for filopodia); *P ≤ 0.05, **P ≤ 0.01 and **P = 0.0006. Source data

To examine further the cellular mechanism behind the cognition phenotype, we analyzed dendritic spines of CA1 and DG hippocampal neurons in P60 female mice (Fig. 7). The hippocampus is a structure important for cognitive functions and is innervated by BF cholinergic neurons^27^, while spine plasticity has a major role in cognitive functions^43,44^. Immature spines are thinner and form fewer stable synaptic contacts than mature mushroom-shaped spines^43,44^ (Fig. 7g), while spine density provides an estimate of synapse density^45^. More specifically, we analyzed segments of secondary dendrites of pyramidal neurons located at the level of the stratum radiatum (Fig. 7a) and found that these spines in *Arg1*-cKO female animals are notably longer and narrower than those in *Arg1*-control animals (Fig. 7c). Morphological analysis of the pyramidal spines showed that P60 *Arg1*-cKO females have an increased proportion of immature spines (filopodia and long, thin spines) and a decreased proportion of mature spines (branched; Fig. 7e). Analysis of granule cell dendritic spines of the outer third of the suprapyramidal blade (Fig. 7b) indicated that, similar to the pyramidal spines, secondary granule cell dendrites in P60 *Arg1*-cKO females were notably narrower (Fig. 7d) and had a higher percentage of immature and lower percentage of mature spines (Fig. 7f).

### *Arg1* microglial deletion impacts long-term potentiation (LTP)

**Fig. 8:**
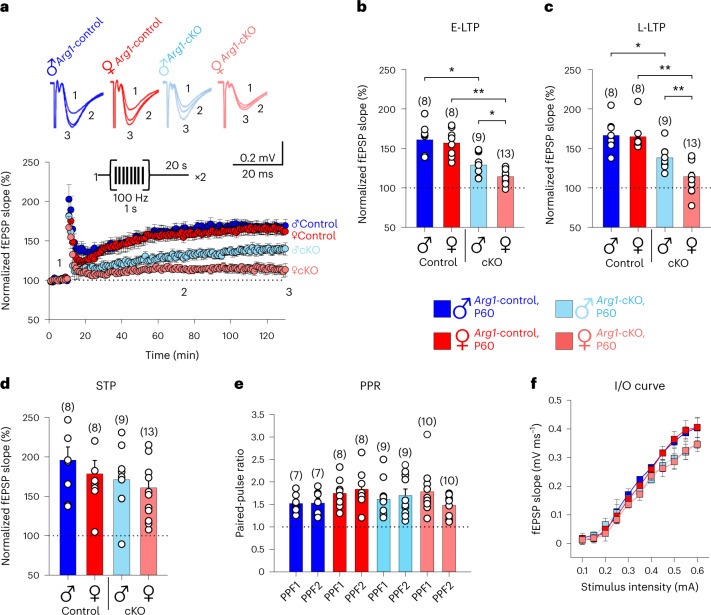
LTP is prevented in 2- to 3-month-old female *Arg1*-cKO mice. **a**, Time course of fEPSPs before and after LTP induction in male *Arg1*-control (blue circles; *n* = 8), female *Arg1*-control (red circles; *n* = 8), male *Arg1*-cKO (light blue circles; *n* = 9) and female *Arg1*-cKO (light red circles; *n* = 13) mice. The inset traces show fEPSPs before (1) and 60 min (2) and 120 min (3) after the plasticity protocol. *Arg1-*control male: 161 ± 7% (E-LTP) and 167 ± 7 % (L-LTP); *Arg1-*control female: 157 ± 6% (E-LTP) and 165 ± 6% (L-LTP); *Arg1*-cKO male: 129 ± 6% (E-LTP) and 139 ± 6% (L-LTP); *Arg1*-cKO female: 114 ± 3% (E-LTP) and 112 ± 4% (L-LTP). **b**,**c**, Histograms show a summary of the results for E-LTP (**b**) and L-LTP (**c**). E-LTP, *Arg1*-control male versus *Arg1*-cKO male, *P* = 0.003; E-LTP, *Arg1*-control female versus *Arg1*-cKO female, *P* < 0.001; E-LTP, *Arg1*-cKO male versus *Arg1*-cKO female, *P* = 0.015; L-LTP, *Arg1*-control male versus *Arg1*-cKO male, *P* = 0.006; L-LTP, *Arg1*-control female versus *Arg1*-cKO female, *P* < 0.001; L-LTP, *Arg1*-cKO male versus *Arg1*-cKO female, *P* < 0.001. **d**, Short-term synaptic potentiation (STP) is not affected in any of the groups studied. *Arg1*-control male: 196 ± 17%, *n* = 8; *Arg1*-control female: 178 ± 17%, *n* = 8; *Arg1*-cKO male: 171 ± 16%, *n* = 9; *Arg1*-cKO female: 161 ± 14%, *n* = 13. **e**, PPR summary data. *Arg1*-control male: 1.53 ± 0.1 after LTP versus 1.52 ± 0.08 at baseline, *n* = 7; *Arg1*-control female: 1.84 ± 0.15 after LTP versus 1.75 ± 0.12 at baseline, *n* = 8; *Arg1*-cKO male: 1.69 ± 0.14 after LTP versus 1.61 ± 0.13 at baseline, *n* = 9; *Arg1*-cKO female: 1.49 ± 0.08 after LTP versus 1.78 ± 0.16 at baseline, *n* = 10; PPF, paired-pulse facilitation. **f**, Input–output (I/O) curves for *Arg1*-control male (*n* = 6), *Arg1*-control female (*n* = 6), *Arg1*-cKO male (*n* = 6) and *Arg1*-cKO female (*n* = 6) mice. The number of slices is shown in parentheses. Data are shown as mean ± s.e.m. Statistically significant differences were determined by unpaired two-sided *t*-tests, with *P* values corrected using the Bonferroni method for multiple comparisons; *P < 0.05 and **P < 0.01. Source data

Thereafter, we wanted to determine whether female *Arg1*-cKO mice induced and expressed LTP at Schaffer collateral–CA1 synapses. We performed a standard induction protocol consisting of two train stimuli of 100 Hz for 1 s separated by 20 s, which elicited robust LTP in female *Arg1*-control mice 60 min and 120 min after the induction protocol (Fig. 8a), and impaired early LTP (E-LTP) and late LTP (L-LTP; Fig. 8b,c) was found in *Arg1*-cKO female mice. Male *Arg1*-cKO mice also exhibited reduced, but not impaired, E-LTP and L-LTP (Fig. 8b,c).

To analyze the short-term potentiation for the different mice used, the means of the first sweeps after applying the plasticity protocol in each experiment were analyzed. Our data indicate that short-term potentiation in the Schaffer collateral–CA1 pathway is not affected in any of the groups studied (Fig. 8d).

To determine the site of expression of LTP, we analyzed paired-pulse facilitation ratios (PPRs) at baseline and 120 min after the application of the induction protocol. The analysis of PPRs before and after LTP did not show differences between animal groups, suggesting that this form of LTP is postsynaptically expressed (Fig. 8e). Finally, to investigate if the basal synaptic transmission is altered, a stimulus–response curve (0.1–0.6 mA; mean of five field excitatory postsynaptic potentials (fEPSPs) at each stimulation strength) was compiled. No differences between groups were found. These data indicate that the differences observed in LTP magnitude between all the groups of mice are not due to a defect in basal synaptic transmission (Fig. 8f).

## Discussion

Compiling data show that microglia are not a homogenous population that respond stereotypically to extrinsic stimuli but instead are a heterogeneous cell type that exhibit distinct transcriptomic profiles^14–18^ and ultrastructural^46^ differences, a diversity that is especially pronounced during early postnatal development (reviewed in ref. ^8^). Here, we report a microglial subtype morphologically indistinguishable from neighboring microglia, which exhibits a substantially different transcriptome, dynamic spatiotemporal localization and functional specialization. We show that the enzyme ARG1 is highly expressed in this microglial subtype and is essential for proper brain development. We provide compelling evidence for a critical role of ARG1^+^ microglia in shaping neuronal circuits involved in cognition. Supporting this, microglial *Arg1* knockout results in impaired neuronal plasticity and cognitive deficits in mice. Previous studies have shown that whole-body *Arg1* knockout in mice is postnatally lethal and causes neurotoxicity^35^. In humans, ARG1 deficiency is a rare autosomal disease^35,36^. As in mice, ARG1-deficient individuals show neurological problems evidenced by progressive neurological and cognitive impairment leading to various degrees of intellectual disability^35^. Most interestingly, the vast majority of individuals whose peripheral symptoms can be managed through diet or drug therapy still continue to suffer from cognitive deficits^35^. Whether human pathology is linked specifically to ARG1 deficiency in microglia requires further investigation.

Our RNA-seq analysis showed robust expression of typical homeostatic genes, including, among others, *Csf1r*, *Cst3*, *Cx3cr1*, *Hexb*, *P2ry12*, *Sparc*, *Tmem119* and *Siglech*^14,31–33,47,48^, indicating that our cell sorting strategy was highly specific for isolating microglia. Recent extensive transcriptomic analyses of microglia have identified several microglia subsets during early postnatal development^14,16,17,22^. One microglia subset was originally identified by high expression of CD11c and insulin-like growth factor 1 and was associated with the corpus callosum and cerebellar white matter^22^. Subsequent studies using transcriptomic analysis of microglia at the single-cell level confirmed the existence of this microglia subtype, which was further defined as tract-associated microglia^17^ or proliferative region-associated microglia^16^. Intriguingly, this microglial subtype was characterized by downregulation of homeostatic microglia markers along with upregulation of several genes typically found under disease conditions (disease-associated microglia)^16,17,32,33^, thus sharing some molecular features with the ARG1^+^ microglia subtype. However, tract-associated microglia do not upregulate the expression of *Arg1* (refs. ^16,17^), which, together with the high enrichment of this microglia subtype in axon tracts and their amoeboid morphology, fully contrast with the phenotype observed in the ARG1^+^ microglial subtype^16,17^. We may wonder how this microglial subtype has escaped identification from most transcriptomic studies performed during postnatal development. One possible explanation is that ARG1^+^ microglia present both spatial and temporal aspects, and their numbers remained limited compared to the overall microglial cell population. Stevens and colleagues^17^ recently identified a small microglia cluster (cluster 1 (the smallest identified cluster in their study) comprising about 0.5% of total microglia), which displays strong upregulation of *Arg1*. The experimental design of the two studies present differences, including the age of animals used (P4/P5 versus P13), genetic background (C57BL/6J versus 129S4/SvJae), area where cells were isolated (no specific location versus the BF), cell isolation protocol and sequencing technology. Yet, the two cell populations display a strong upregulation of genes such as *Apoe*, *C3*, *Lgals3*, *Mgl2* and *Igf1* (Extended Data Fig. 7g) and are more prominent during early postnatal stages^17^, thus validating the ARG1^+^ microglial subtype independently. In addition, the cluster 1 size shows sex-specific variation, with enrichment in female mice^17^. This observation is important as it provides strong support to the expanding literature about sex differences in microglia^23,24^ and it suggests directions for future research to understand differences in the behavioral studies we observed in this study. Although here we report ARG1^+^ microglia in both sexes that exist in equal numbers (Extended Data Fig. 6) and have the same transcriptomic profiles (Extended Data Fig. 5b), this contradiction only highlights that a systematic analysis of microglial phenotypes needs to be performed to understand how microglial subtypes contribute to physiology and disease of the brain^8,9^. Taken together, we can conclude that ARG1^+^ microglia are a developmentally regulated subtype that display a distinctive molecular signature and have a specific function in shaping the brain.

An outstanding observation of the present study was the highly enriched presence of ARG1^+^ microglia within the BF of the developing brain, where main cholinergic groups are located and known to play major roles in cognitive processes, such as attention, learning and memory^49,50^. Remarkably, we found substantial impairment in long-term memory in females but not in males lacking *Arg1* in microglia, thus sustaining a sexual dimorphism related to the ARG1^+^ microglia subtype. Importantly, motor behavior was not affected in *Arg1*-cKO mice, therefore raising the possibility that forebrain cholinergic dysfunction underlies the cognitive deficits in female mice lacking *Arg1*. Notably, the appearance of ARG1^+^ microglia mimicked the temporal maturation of main cholinergic markers, including ChAT and acetylcholinesterase expressions and activities, which rise progressively until reaching adult levels between the third and fourth postnatal week^50,51^. Additionally, dendritic growth and branching, along with the increase in perikaryal size, starts within the first 3 postnatal weeks to reach adult levels by P30 (ref. ^51^), again fully coincident with the appearance of ARG1^+^ microglia in the BF. Our immunohistochemical and ultrastructural analysis demonstrated the interaction between cholinergic neurons/processes and ARG1^+^ microglia, and, hence, the possibility that this microglial subtype contributes to the maturation of the BF cholinergic system is certainly plausible. Supporting this, a deficient cholinergic innervation to the hippocampus was evident in *Arg1*-cKO female mice, a critical process given the vital role of cholinergic innervation in the regulation of synaptic communication and plasticity within the hippocampus^52^. During development, spines mature from a thin, elongated shape to a mushroom-like structure^53^, a process critically involved in LTP^54^. We provide compelling evidence that both forms of synaptic plasticity are largely affected in the hippocampi of *Arg1*-cKO female mice, thus raising the importance of this microglia subtype in regulating critical aspects of hippocampus-dependent long-term memory. First, our morphological analysis revealed that pyramidal and dentate spines showed an increased proportion of immature spines and a decreased proportion of mature spines in females. It is important to highlight that dendritic spines represent the main unitary postsynaptic compartment for excitatory input, the basis for LTP induction^53^. Among the different forms of synaptic plasticity, that relying on NMDA receptor-dependent LTP in the CA1 region of the hippocampus is probably the most studied given its close correlation with learning and memory^53^. In turn, LTP and behavioral paradigms, such as spatial learning memory, are tightly associated processes^55^. Given the striking sexual dimorphism observed for the ARG1^+^ microglial subtype, we wanted to know how male and female *Arg1*-cKO mice induced and expressed LTP at Schaffer collateral–CA1 synapses. The electrophysiological analysis revealed that *Arg1* is required for long-term plasticity in both male and female mice, therefore substantiating our RNA-seq transcriptional analysis showing similar molecular signatures from ARG1^+^ microglia in both sexes. However, a substantial functionally relevant difference was observed in terms of requirement between males and females. While LTP was present but reduced in magnitude in *Arg1*-cKO males, the induction of LTP in *Arg1*-cKO females was impaired. These results indicate a different requirement of microglial *Arg1* for males and females, being critical for females. By analyzing PPR, we confirmed the postsynaptic locus of this form of LTP as previously established^56^. Interestingly, and consistent with this, short-term plasticity was not altered in any genotype. By studying the characteristics of input–output curves, no differences were found between different genotypes. These results indicate that the differences found in LTP are not due to changes in the presynaptic machinery of release. In fact, changes were found postsynaptically in the distribution-type of dendritic spines between male and female mice. However, we cannot exclude the possibility that phenotypes observed in the hippocampus are due to a direct impact from local microglia (for example, through low but biologically important *Arg1* mRNA expression by these cells), we did not observe ARG1^+^ microglia in this area. In conclusion, electrophysiological results fully agree with the changes observed in (1) dendritic spine maturation in the hippocampus and (2) behavioral results demonstrating that a disruption of LTP in *Arg1*-cKO females coincides with defects in long-term memory in the same mice.

Previous reports have identified intrinsic roles of early postnatal microglia in synaptic stripping and pruning, modulation of synaptic transmission and synaptogenesis, apoptotic cell corpse removal and neuron survival^7,8^. In this study, we have identified ARG1^+^ microglia, morphologically indistinguishable from ARG1^–^ microglia, which emerge as a highly dynamic subtype involved in elaboration and maturation of the BF cholinergic system and hippocampal synaptic plasticity in female mice. Maturation of this system is of critical importance in developmental disorders, including autism^50^, and Alzheimer’s disease^57^. Of note, the involvement of microglia in Alzheimer’s disease pathology is highly recognized in the field^58^, and two-thirds of individuals suffering from Alzheimer’s disease are women, whose underlying mechanism is not explained by only differences in longevity^59^. Our study provides a step toward understanding how ARG1^+^ microglia regulate the development of the BF and highlights the potential involvement in pathological conditions.

## Methods

### Animals

All experimental animal protocols in the present study were in accordance with the respective national, federal and institutional regulations, that is, the Guidelines of the European Union Council, following Swedish regulations for the use of laboratory animals and approved by the Regional Animal Research Ethical Board, Stockholm, Sweden (ethical permits N248/13), and the Scientific Committee of Instituto de Investigación y Formación Agraria y Pesquera, Consejería de Agricultura, Pesca, Agua y Desarrollo Rural of Junta de Andalucía (Spain; 03/05/2018/069) and in conformity with the Canada Council on Animal Care guidelines. C57BL/6J (WT; Charles River), YARG (The Jackson Laboratory, stock 015857), *Cx3cr1*^GFP^(The Jackson Laboratory, stock 005582), *Arg1*^fl/fl^ (The Jackson Laboratory, stock 008817) and *Cx3cr1*^CreER^ (The Jackson Laboratory, stock 021160) mice were used and maintained under a 12-h light/12-h dark cycle at 22–25 °C with access to food and water ad libitum.

#### Generation of microglia-specific ARG1-deficient mice (*Arg1*-cKO)

*Arg1*^fl/fl^ mice with the *Arg1* allele bearing *loxP* sites flanking exons 7 and 8 were crossed with *Cx3cr1*^CreER^ mice to generate *Arg1*^fl/fl^*Cx3cr1*^CreER+/−^ (*Arg1*-cKO) and *Arg1*^fl/fl^*Cx3cr1*^CreER−/−^ (*Arg1*-control) mice (Fig. 5a). The deletion was induced after daily tamoxifen treatment for 2 or 3 consecutive days starting at P1 (Fig. 5b). All mice (Cre^+^ and Cre^–^) were injected with tamoxifen at a dose of 250 μg per pup. Genotyping of the mice was done by PCR analyses of finger DNA using primers as presented in Supplementary Table 2.

### Tissue preparation, immunohistochemistry and confocal laser microscopy

Animals were deeply anesthetized with sodium pentobarbital, transcardially perfused with 0.9% sodium chloride and fixed with 4% paraformaldehyde in 0.1 M phosphate buffer (pH 7.4). Brains were collected and postfixed in the same fixative for 24 h and transferred to 30% sucrose in 0.1 M phosphate buffer for cryoprotection for a minimum of 3 d. Brains were cryosectioned using a sliding microtome (Leica, SM2000R) into 25-µm free-floating sections and stored as 1:12 series at 4 °C. When necessary, antigen retrieval was performed by heat-induced antigen retrieval, followed by PBS washes, permeabilization with 0.3% Triton X-100 in PBS and blocking with 0.3% Triton X-100 and 10% donkey serum in PBS. Sections were washed in PBS before incubation with secondary antibodies (Alexa Fluor). Sections were washed in PBS, counterstained with Hoechst or DAPI, further washed in PBS and mounted in Fluoromount-G.

For DAB staining, brain sections were deparaffinized and hydrated through treatment with xylenes and a graded alcohol series, followed by treatment with 0.6% hydrogen peroxide in TBS. Sections were washed in TBS, permeabilized in TBS Tween 20 (0.1%), blocked with TBS Tween 20 (0.1%) and 5% normal serum and incubated with primary antibody in blocking solution overnight at 4 °C. Sections were washed with TBS and incubated with SignalStain Boost detection reagent and SignalStain DAB. For information about antibodies, reagents and hardware and software used, see Supplementary Table 2.

#### Manual cell number quantification

Microglia and ChAT^+^ somas were identified based on corresponding markers. The numbers of animals used are denoted by circles, squares and/or triangles.

#### Intensity analyses

The mean intensity of the fluorescent marker CD68 was measured by performing mask outlining of the ARG1 area using the same value of threshold in each image, followed by automatic measurement of the mean intensity inside these areas using Fiji ImageJ software^60^. The percentage of ChAT labeling in the female CA1 was determined automatically by defining outline masks based on brightness thresholds with Fiji software.

#### Morphometric analysis

Individual microglia from the BF/vStr, with their nuclei in the center of the *z* plane, were selected. Three-dimensional confocal *z* stacks were automatically reconstructed using a self-customized Python-based script^61^. Reconstructions were visually checked using the ImageJ plugin ‘simple neurite tracer’. Each cell was individually extracted, and the number of branches, path length, branch order and process volume were quantified using the open-source software L-measure. Four mice per developmental stage were examined. At least 15 (P10) and 32 (P28) microglia of each phenotype were analyzed. For information about antibodies and hardware and software used, see Supplementary Table 2.

#### Golgi–Cox staining

Segments of secondary dendrites of pyramidal neurons located at the level of the stratum radiatum (CA1) and granule cell dendritic spines of the outer third of the suprapyramidal blade (DG) were considered for quantitative analysis. Dendrite segments from 15 to 20 µm in length filled by the mercuric reaction of the Golgi–Cox method were used.

### iDISCO+

#### iDISCO+ immunostaining and tissue clearing

iDISCO+ immunostaining and tissue clearing was performed as previously published^26,62^. P10 and P28 WT male mouse brains were perfused using 4% paraformaldehyde and fixed overnight at 4 °C. Brains were washed with PBS, cut into half hemispheres, treated with an up-series of methanol solutions and stored at −20 °C. Samples were treated with 5% hydroxyperoxide in methanol overnight at 4 °C, treated with a reverse concentration of methanol solution and washed with iDISCO washing buffer. After permeabilization and blocking, samples were incubated with primary antibodies for 5–7 d at 37 °C with gentle rotation. Samples were then washed with iDISCO washing buffer for 1 d and incubated with secondary antibodies for 5–7 d at 37 °C. Samples were washed with iDISCO wash buffer (1 d) with gentle rotation at 37 °C. The immunostained hemispheres were treated with an up-series of methanol solution. Samples were incubated in 33% methanol and 66% dichloromethane for 3 h and 100% dichloromethane for 15 min twice and were transferred in dibenzyl ether (DBE). After 1 d of incubation with DBE, the DBE solution was changed to fresh solution and incubated for 1 d before imaging. For information about antibodies and reagents used, see Supplementary Table 2.

#### iDISCO+ imaging acquisition

Cleared brain images were acquired with a COLM microscope^63^. The hemisphere was placed in a quartz cuvette filled with DBE, and the position was fixed using silicone blocks. Immunostaining signal was acquired with a 647- or 561-nm channel, and autofluorescence was assessed with a 488- or 405-nm laser channel for image registration. The original image resolution was 0.585, 0.585 and 5 μm in the *x*, *y* and *z* axes. For information about materials used, see Supplementary Table 2.

#### iDISCO+ image analysis

Original TIFF files (16 bit) were downsampled to an isotropic resolution of 5 μm and converted into 8 bit by custom MATLAB script and Fiji^64^. Each *z*-stack image was stitched by TeraStitcher^65^. The stitched images were processed by a series of processing filters (unsharp mask, background subtraction, integral filter and fast Fourier transform filter) in ImageJ and Amira 3D software (Thermo Fisher Scientific). ARG1^+^ and IBA1^+^ cells were recognized by signal intensity and morphology. After removing blood vessels manually in Amira segmentation editor, ARG1^+^ and IBA1^+^ cells were segmented by global threshold segmentation. Segmented images were labeled, counted and converted into a points cloud by Amira. Movies were generated using Amira.

#### Image registration

Image registration was performed using Elastix toolbox^66,67^ and MelastiX MATLAB wrapper (https://github.com/raacampbell/matlab_elastix). Four different methods of image registration (Rigid, Similarity, affine and B-Spline) were sequentially performed to register sample data. First, we acquired reference brain images (P28 and P10) stained with Neurotrace 640/660 (N21483, Thermo Fisher Scientific). Second, we transformed the reference brain image to Allen Brain Atlas (developing mouse, P56 and P14, June 2013 v.2) using the Neurotrace 640/660 channel and obtained an autofluorescent image registered to Allen Brain Atlas (AutoF Allen brain atlas). Third, immunostained brain images were registered to the AutoF Allen Brain Atlas using the autofluorescence channel of the immunostained brain.

### Electron microscopy

#### Immunoperoxidase staining for electron microscopy

Three YARG P13 female pups were anesthetized with a mixture of ketamine and xylazine (80 and 10 mg per kg (body weight), intraperitoneal) and were transcardially perfused with PBS (50 mM, pH 7.4), followed by 3.5% acrolein and 4% paraformaldehyde. Brains were cut in ice-cold PBS and stored at −20 °C in cryoprotectant until further processing^68^. Brain sections selected in the BF (bregma 0.38 mm to 0.02 mm) were rinsed in PBS, incubated in 0.1 M citrate buffer for 40 min at 70 °C for antigen retrieval and quenched with 0.3% hydrogen peroxide for 5 min, followed by treatment with 0.1% sodium borohydride for 30 min. Sections were washed in PBS and blocked in 10% donkey serum with 0.03% Triton X-100 in PBS for 1 h at room temperature and were incubated overnight with anti-ARG1. The next day, sections were rinsed in TBS (50 mM, pH 7.4) and incubated with a secondary antibody conjugated to biotin and with a Vectastain avidin–biotin complex staining kit (Vector Laboratories). Sections were developed in a Tris buffer solution (50 mM, pH 8.0) containing 0.05% diaminobenzidine and 0.015% hydrogen peroxide and rinsed with phosphate-buffered solution (100 mM, pH 7.4). Sections were postfixed with 1% osmium tetroxide, dehydrated using sequential alcohol baths, treated with propylene oxide and embedded in Durcupan resin between fluoropolymer sheets at 55 °C for 3 d, as described previously^69^. After ultrathin section generation, microglia positive and negative for ARG1 were imaged and photographed. For information about antibodies, material, reagents and hardware used, see Supplementary Table 2.

#### Ultrastructural analysis

Ultrastructural analysis was performed using QuPath software (10–11 ARG1^+^ cells and 9–11 ARG1^–^ cells per animal; *n* = 3 animals). Microglial contacts with cell bodies belonging to other brain cells and compartments, including blood vessels, were quantified. Microglia were identified by their heterogenous euchromatin and heterochromatin pattern, electron-dense cytoplasm and distinctive organelles, such as their frequent long stretches of endoplasmic reticulum and lipidic inclusions (that is, lysosomes and lipid droplets)^70,71^. Microglia were classified as ARG1^+^ or ARG1^–^ depending on the presence (cytoplasmic electron-dense peroxidase precipitate) or absence of immunohistoreactivity against ARG1 (complete absence or faint peroxidase precipitate near the cytoplasmic membrane while most of the cytoplasm remained unstained).

Neuronal cell bodies were identified by their electron-lucent cytoplasm and nuclei, often with distinct nucleoli, together with the presence of apical dendrites and axon terminal innervation. Axon terminals were identified by the presence of synaptic vesicles, while dendritic spines were positively identified based on their contact with an axon terminal and the presence of a postsynaptic density. A contact with a synaptic cleft was considered when microglia simultaneously contacted both the axon terminal and dendritic spine forming a synapse^70^. Astrocytic cell bodies were identified by their electron-lucent nuclei characterized by a heterochromatin pattern with a thin electron-dense rim along the nuclear membrane and electron-lucent cytoplasm often containing intermediate filaments. Blood vessels were identified by empty space framed by a basement membrane and underlying pericytes and endothelial cells with tight junctions^71^. Contacts with blood vessels were considered when microglial cell bodies were in direct contact with the basement membrane of the blood vessel. Proximity to blood vessels was considered when microglia were contacting an astrocytic process directly adjacent to the basement membrane^70^. Both types of interactions with vessels were analyzed together. Microglial mitochondria, endoplasmic reticulum, Golgi apparatus, lysosomes and phagocytic inclusions were quantified. Mitochondria were classified as holy mitochondria when they presented a circular empty space contained within their membranes^72^. Phagocytic inclusions were classified by the nature of their contents, that is, empty if the interior of the inclusion was clear, with an axon terminal when synaptic vesicles were identified within the inclusion and with content if the vesicle had content of another nature. Lysosomes were identified by their dark color with heterogenous content contained within a single membrane. Primary and secondary lysosomes were discriminated by the presence of fused endosome vesicles in secondary lysosomes versus an absence in primary lysosomes^71,73,74^. Nuclear indentations were identified by an invagination of the nuclear membrane^71,75^.

### RNA-seq and RT–qPCR

#### Microglia isolation

Brains from three to five P13 YARG female or male mice were perfused with 10 ml of cold PBS. The olfactory bulb was removed, and the two hemispheres were separated. Next, the median plane of each hemisphere was placed facing upward, a cut was made posterior to the lateral ventricle, and the tissue ventral to the corpus callosum (including the cerebral nucleus) was dissected out. Tissues were pooled, minced with a scalpel, further dissociated with mechanical dissociation using a tissue grinder on ice (for RNA-seq analysis) and thereafter filtered through 70-µm nylon mesh. The microglial fraction was enriched by a 20% Percoll density gradient at 4 °C. For information about reagents used, see Supplementary Table 2.

#### FACS

The microglial population was initially gated based on size and granularity, followed by gating for singlets, negative selection with CD206–BV421 to exclude macrophages and positive selection with CX3CR1–APC. Finally, microglia were sorted to ARG1^*+*^ and ARG1^–^ based on YFP expression using a FACSAria III cell sorter system and analyzed using FACSDiva software (BD Biosciences). Cell populations (ARG1–YFP^+^CX3CR1^+^CD206^–^, ARG1–YFP^−^CX3CR1^+^CD206^−^ and CD206^+^) were collected directly in Qiazol (Qiagen). For information about antibodies used, see Supplementary Table 2.

#### RNA isolation and RT–qPCR

RNA was isolated using commercial kits (RNeasy micro). cDNA was synthesized using oligo d(T), dNTPs and Superscript III (Invitrogen). RT–qPCR was performed using a StepOne Plus instrument (Applied Biosystems) with SYBR Green master mix (Life Technologies) and predesigned primers (KiCqStart Primers, Sigma). Relative gene expression levels were normalized to *Actb* in each sample with the ΔΔ*C*_*t*_ method. For information about reagents used, see Supplementary Table 2.

#### Library preparation and RNA-seq

cDNA was prepared using a SMART-Seq v4 Ultra Low Input RNA kit for sequencing (Takara Bio, 634898). The cDNA quality was examined on an Agilent TapeStation system using a High-Sensitivity D5000 ScreenTape (Agilent, 5067-5592). One nanogram of cDNA was used for library preparation using a Nextera XT DNA library preparation kit (Illumina, FC-131-1024 and FC-131-1096). The yield and quality of the amplified libraries were analyzed using Qubit (Thermo Fisher) and the Agilent TapeStation. The indexed cDNA libraries were normalized and combined, and the pools were sequenced on an Illumina NextSeq 550 for a 75-cycle v2 sequencing run generating 75-base pair single-end reads.

#### RNA-seq data and computational analysis

Basecalling and demultiplexing were performed using Illumina bcl2fastq v2.20.0 software with default settings, generating fastq files for further downstream mapping and analysis. Reads were aligned to the Ensembl GRCm38/mm10 reference genome using STAR v2.6.1d. Gene counts were estimated using featureCounts (v1.5.1). Normalization and sample group comparisons of gene counts were performed using the R package DESeq2 (v1.28.1). No filtering was performed before sample group comparisons, where the default DESeq2 independent filtering was applied. For the ‘positive versus negative’ comparison, a volcano plot was created in GraphPad Prism, which displayed significance and FC for the dataset together with gene symbols for the most highly regulated genes. A heat map for validated genes found to be differentially expressed (up- or downregulated by at least twofold) between the two microglial populations of interest was generated using GraphPad Prism.

### Behavior tests

The experimental mice were subjected to the following described series of behavioral paradigms.

#### Open field

The open field test was used to assess both exploratory behavior and locomotor activity. Mice were placed for 5 min in an open field (45 × 45 × 45 cm^3^). Monitoring was performed with an automated tracking system (SMART 2.5, Panlab). The behavioral parameters registered during 5-min sessions were the percentage of distance travelled in border and center zones, and, to measure a possible anxiety behavior, we calculated the time spent, in percent, in a border zone (*Arg1*-control, *n* = 10 females and *n* = 8 males; *Arg1*-cKO, *n* = 9 females and *n* = 6 males).

#### Object recognition memory

The object recognition task was used for assessing recognition memory, taking advantage of the ability to discriminate the familiarity of previously met objects. Mice were tested as described previously^76^. Briefly, female mice were placed in a cubic arena (45 × 45 × 45 cm^3^) with two identical objects, which they were allowed to explore for 15 min (training phase). One hour later, animals were exposed again to two objects, one familiar and one novel, for 10 min. The number of approximations to exploring the novel object compared to the number of explorations of the familiar object assessed the animal’s short-term memory (10 min). Twenty-four hours later, animals were exposed again to two objects, a familiar one and a new one, for 10 min, assessing the animal’s long-term memory. The relative exploration of the novel object was expressed as a discrimination index (number novel – novel familiar)/(number novel + number familiar), taking into account the training index (*Arg1*-control, *n* = 10; *Arg1*-cKO, *n* = 8).

#### Y maze

The Y maze is a test to investigate spatial memory. The maze was made of methacrylate, and each arm was 18 cm long, 38 cm high and 8 cm wide and positioned at equal angles. Working memory was assessed by recording spontaneous exploring behavior in a Y maze. The mice were placed in the center of the maze and allowed to freely explore for 9 min. In this training, we measured the spontaneous alternation triplet and the total number of entries in each arm. To study spatial memory, we used the novel arm discrimination task based on the innate preference of rodents to explore a novel environment more than a familiar one. Therefore, we blocked a specific arm for 5 min. After 1 h, the animals were placed again in the maze with all three arms opened and allowed to explore the familiar arms and the novel arm for 4 min. The percentage of entries in each arm in relation to the percentage of the first session was scored. The whole session was recorded by video and analyzed later using SMART 2.5, Panlab (*Arg1*-control, *n* = 11 females and *n* = 8 males; *Arg1*-cKO, *n* = 9 females and *n* = 6 males).

#### Rotarod

The rotarod was used to assess motor learning and neuromuscular coordination. To habituate mice to the rotarod (Ugo Basile Biological Research Apparatus), the animals were placed on the roller at a speed of 20 r.p.m. until they could remain on it for 1 min without falling off. To assay motor coordination, mice were tested as described previously^77^. Briefly, animals were tested at a rotational speed of 20 r.p.m., accelerating to 60 r.p.m. in increments of 5 r.p.m. during four successive trainings and quantifying the latency of the first fall and the number of total falls. Finally, the next day, the protocol was repeated as a final test to estimate the motor memory in all experimental groups (*Arg1*-control, *n* = 10 females and *n* = 8 males; *Arg1*-cKO, *n* = 9 females and *n* = 6 males).

### Electrophysiology

For electrophysiological recordings, 2-month-old male and female mice were used.

#### Slice preparation

Hippocampal slices were prepared as described in detail elsewhere^78,79^. Mice were anesthetized with isofluorane (2%) and decapitated for slice preparation. Briefly, after decapitation, the whole brain, containing the two hippocampi, was removed into ice-cold solution (I), positioned on the stage of a vibratome slicer and cut to obtain transverse hippocampal slices (350 mm thick), which were maintained continuously oxygenated for at least 1 h before use. All experiments were performed at near-physiological temperatures (31–33 °C). For experiments, slices were continuously perfused with the solution described above. For composition about reagents used, see Supplementary Table 2.

#### Electrophysiological recordings

fEPSPs were recorded in the CA1 region of the hippocampus and were evoked by a stimulating electrode placed on the Schaffer collaterals (0.2 Hz). Extracellular recording electrodes were filled with solution I. Stimulation was adjusted to obtain an fEPSP amplitude of approximately 0.2 mV under control conditions. In paired-pulse experiments, two consecutive stimuli separated by 40 ms were applied. Data were filtered at 3 kHz and acquired at 10 kHz. A stimulus–response curve (0.1–0.6 mA, mean of five fEPSPs at each stimulation strength) was compiled for the different mice used.

#### Plasticity protocol

After a stable fEPSP baseline period of 10 min, LTP was induced by a protocol consisting of two trains of stimuli at 100 Hz, 1 s separated by 20 s. Recordings lasted 60 and 120 min after the application of the protocol.

#### Data analysis

Data were analyzed using Clampfit 10.2 software (Molecular Devices). The last 10 min of recording were used to estimate changes in synaptic efficacy compared to baseline. The PPR was expressed as the slope of the second fEPSP divided by the slope of the first fEPSP. Data are presented as mean ± s.e.m. Statistical comparisons were made using Student’s *t*-tests. *P* values less than 0.05 were considered statistically significant.

### Statistical analysis

All statistical analyses were conducted using Prism 8 (v.9.3.1, GraphPad Software) and Stata (v 17.0, StataCorp). Results are presented as the mean ± s.e.m. Data normality was tested using the Shapiro–Wilk test. For normally distributed data, differences in means between groups were examined using two-tailed paired or unpaired Student’s *t*-tests or analysis of variance, whichever was appropriate. For data that were not normally distributed, a Wilcoxon signed-rank test for paired data, a Mann–Whitney *U*-test or a Kruskal–Wallis test was used. Differences were considered statistically significant if *P* values were less than 0.05. Raw data are available as Source Data.

### Statistics and reproducibility

All experimental studies are guided by the 3R principle, EU Directive 2010/63/EU. The investigators were blinded to the conditions of the experiments during data collection and quantification. No statistical methods were used to predetermine sample sizes, but these are consistent with previous publications^78–83^. No data points have been excluded. We excluded animals without mobility in both groups. These exclusion criteria were preestablished. Animals were randomly assigned to two groups based on genotype and were further subdivided by gender for behavioral studies. For morphometric and manual counting analyses, sample IDs were randomized using an Excel-generated randomized numerical ID.

### Reporting summary

Further information on research design is available in the Nature Portfolio Reporting Summary linked to this article.

## Online content

Any methods, additional references, Nature Portfolio reporting summaries, source data, extended data, supplementary information, acknowledgements, peer review information; details of author contributions and competing interests; and statements of data and code availability are available at 10.1038/s41593-023-01326-3.

## Supplementary information

Reporting Summary

Supplementary Video 1 iDISCO+ 3D presentation of ARG1^+^ microglia clusters in a P10 WT mouse brain. Each dot represents a single ARG1^+^IBA^+^ cell (*n* = 1 animal).

Supplementary Video 2 iDISCO+ 3D presentation of ARG1^+^ microglia clusters in a P28 WT mouse brain. Each dot represents a single ARG1^+^IBA^+^ cell (*n* = 1 animal).

Supplementary Video 3 iDISCO+ 3D presentation of the main ARG1^+^ microglia cluster in a P10 WT mouse brain (*n* = 3 animals).

Supplementary Video 4 iDISCO+ 3D presentation of the main ARG1^+^ microglia cluster in a P28 WT mouse brain (*n* = 3 animals).

Supplementary Tables Supplementary Table 1: RNA-seq data. Supplementary Table 2: Key resources table.

## Data Availability

The bulk RNA-seq data that support the findings of this study are available at Gene Expression Omnibus under accession number GSE216893. Source data are provided with this paper. All other data are available from the corresponding authors upon request.

## Source data

Source Data Fig. 1 Statistical source data.
Source Data Fig. 2 Statistical source data.
Source Data Fig. 3 Statistical source data.
Source Data Fig. 4 Statistical source data.
Source Data Fig. 5 Statistical source data.
Source Data Fig. 6 Statistical source data.
Source Data Fig. 7 Statistical source data.
Source Data Fig. 8 Statistical source data.
Source Data Extended Data Fig. 3 Statistical source data.
Source Data Extended Data Fig. 6 Statistical source data.
Source Data Extended Data Fig. 7 Statistical source data.
Source Data Extended Data Fig. 8 Statistical source data.
Source Data Extended Data Fig. 9 Statistical source data.
Source Data Extended Data Fig. 10 Statistical source data.
