# Supplementary material for: ARG1-expressing microglia show a distinct molecular signature and modulate postnatal development and function of the mouse brain
Source: Nat Neurosci. 2023 May 11;26(6):1008–20. doi: 10.1038/s41593-023-01326-3 (PMC10244174; doi:10.1038/s41593-023-01326-3)
Supplement: Supplementary file 1 — Reporting Summary [file 41593_2023_1326_MOESM1_ESM.pdf]

nature portfolio

Corresponding author(s): Vassilis Stratoulias, Bertrand Joseph

Last updated by author(s): March 19, 2023

Reporting Summary

Nature Portfolio wishes to improve the reproducibility of the work that we publish. This form provides structure for consistency and transparency in reporting. For further information on Nature Portfolio policies, see our Editorial Policies and the Editorial Policy Checklist.

Statistics

For all statistical analyses, confirm that the following items are present in the figure legend, table legend, main text, or Methods section.

n/a	Confirmed	
☐	☒	The exact sample size (*n*) for each experimental group/condition, given as a discrete number and unit of measurement
☐	☒	A statement on whether measurements were taken from distinct samples or whether the same sample was measured repeatedly
☐	☒	The statistical test(s) used AND whether they are one- or two-sided<br>*Only common tests should be described solely by name; describe more complex techniques in the Methods section.*
☒	☐	A description of all covariates tested
☒	☐	A description of any assumptions or corrections, such as tests of normality and adjustment for multiple comparisons
☐	☒	A full description of the statistical parameters including central tendency (e.g. means) or other basic estimates (e.g. regression coefficient) AND variation (e.g. standard deviation) or associated estimates of uncertainty (e.g. confidence intervals)
☒	☐	For null hypothesis testing, the test statistic (e.g. *F*, *t*, *r*) with confidence intervals, effect sizes, degrees of freedom and *P* value noted<br>*Give P values as exact values whenever suitable.*
☒	☐	For Bayesian analysis, information on the choice of priors and Markov chain Monte Carlo settings
☐	☒	For hierarchical and complex designs, identification of the appropriate level for tests and full reporting of outcomes
☒	☐	Estimates of effect sizes (e.g. Cohen's *d*, Pearson's *r*), indicating how they were calculated

Our web collection on statistics for biologists contains articles on many of the points above.

Software and code

Policy information about availability of computer code

Data collection	- Imaging: ZEN black 2.3 (Zeiss)<br>- iDISCO: Operation software of the COLM is built by custom LabView code (PMID: 24945384)<br>- qPCR: StepOne (ThermoFisher Scientific)<br>- FACs: FACSDiva™ software (BD Biosciences)<br>- Behavioral studies: SMART 2.5 (Panlab)<br>- Electrophysiological recordings: Clampfit 10.2 (Molecular Devices)
Data analysis	- Image analysis and figure assembly: ImageJ/Fiji 1.53t, Photoshop (Adobe), CorelDraw (Corel).<br>- Electron microscopy: QuPath<br>- FACS analysis: FACSDiva™ software (BD Biosciences).<br>- iDISCO: RStudio, MATLAB, TeraStritcher (PMID: 23181553), Amira 3D software (ThermoFisher Scientific), Elastix toolbox (PMID: 19923044, PMID: 24474917), MelastiX MATLAB wrapper (https://github.com/raacampbell/matlab_elastix), Adobe Premiere, GIMP, Inkscape, Microsoft Excel.<br>- Morphometric analysis (microglia): Microglial tri-dimensional reconstructions were done with a self-customized script (Altivie F, et al.2018). The script was developed with Python 4.5.<br>- Dendritic spine analysis: ImageJ, Reconstructor (Texas University).<br>- Basecalling and demultiplexing was performed using Illumina bcl2fastq v2.20.0 software. Reads were aligned to Ensembl GRCm38/mm10 reference genome using STAR v2.6.1d. Gene counts were estimated using featureCounts (v1.5.1). Normalization and sample group comparisons of gene counts were performed using R package DESeq2 (v1.28.1). No filtering was performed prior to sample group comparisons, where the default DESeq2 independent filtering was applied.

- Statistical analysis and graph generation: Prism 8 (v.9.3.1) (GraphPad Software, San Diego, CA, USA) and Stata (v 17.0, StataCorp, College Station, TX, USA).

For manuscripts utilizing custom algorithms or software that are central to the research but not yet described in published literature, software must be made available to editors and reviewers. We strongly encourage code deposition in a community repository (e.g. GitHub). See the Nature Portfolio guidelines for submitting code & software for further information.

Data

Policy information about availability of data

All manuscripts must include a data availability statement. This statement should provide the following information, where applicable:

- Accession codes, unique identifiers, or web links for publicly available datasets
- A description of any restrictions on data availability
- For clinical datasets or third party data, please ensure that the statement adheres to our policy

The data supporting the findings of this study are available within the article, the Supplementary Information files and the Source Data files that accompany this article. RNA-Seq data are deposited in the Gene Expression Omnibus (GSE216893). Any additional data relevant to the manuscript are available from the authors upon request.

Human research participants

Policy information about studies involving human research participants and Sex and Gender in Research.

Reporting on sex and gender	N/A
Population characteristics	N/A
Recruitment	N/A
Ethics oversight	N/A

Note that full information on the approval of the study protocol must also be provided in the manuscript.

Field-specific reporting

Please select the one below that is the best fit for your research. If you are not sure, read the appropriate sections before making your selection.

☒ Life sciences ☐ Behavioural & social sciences ☐ Ecological, evolutionary & environmental sciences

For a reference copy of the document with all sections, see nature.com/documents/nr-reporting-summary-flat.pdf

Life sciences study design

All studies must disclose on these points even when the disclosure is negative.

Sample size	No statistical methods were used to predetermine sample sizes, but our sample sizes are similar to those reported in previous publications (PMIDs: 32873805, 30169759, 31006066, 35493109, 26358557, 30104733).
Data exclusions	For behavioural studies we excluded animals without mobility in both groups. This exclusion criteria were pre-established. No other data were excluded from these analyses.
Replication	Data are representative of multiple independent experiments. For each representative image, experiments were performed at least three times with similar results.
Randomization	For behavioural studies, the animals were randomly selected and separated into two groups according to the genotype. In addition, they were subdivided according to gender. For morphometric and manual counting analyses, sample IDs were randomized using randomized numerical ID in Excel (Microsoft).
Blinding	All behavioural studies were blinded including the analysis of the recollected data. For cell manual counting (V.S., R.R., M.C., P.G.R.), morphometric (D.T.), dendritic spine analysis (E.M.P.V.) and electrophysiological analysis (I.M.G.), respective investigators performing the analysis were blinded to the genotypes during data collection.

Reporting for specific materials, systems and methods

We require information from authors about some types of materials, experimental systems and methods used in many studies. Here, indicate whether each material, system or method listed is relevant to your study. If you are not sure if a list item applies to your research, read the appropriate section before selecting a response.

Materials & experimental systems

n/a	Involved in the study
☐	☒ Antibodies
☐	☐ Eukaryotic cell lines
☐	☐ Palaeontology and archaeology
☐	☒ Animals and other organisms
☐	☐ Clinical data
☐	☐ Dual use research of concern

Methods

n/a	Involved in the study
☐	☐ ChIP-seq
☐	☒ Flow cytometry
☐	☐ MRI-based neuroimaging

Antibodies

Antibodies used

- ARG1 (Abcam, ab91279, rabbit polyclonal, Lot#GR3248025-1, GR302764-1); RRID:AB-10674215.
- ARG1 (SantaCruz, sc-18354, goat polyclonal, Lot#J2015); RRID:AB_2227469.
- ARG1 (SantaCruz, sc-271430, clone E-2, Lot#B0717); RRID:AB_10648473.
- CD206 (R&D, AF2535, goat polyclonal, Lot#ABTU0315101); RRID:AB_ 2063012.
- CD206-BV421 (Biolegend, 141717, clone C068C2, Lot#B218581); RIDD:AB_2562232.
- CD68 (Thermo Fisher Scientific, 14-0681-82, rat monoclonal); RIDD:AB_2572857.
- ChAT (Sigma-Aldrich, MAB5350, mouse); RRID:AB_95218.
- CLEC7A (Invivogen, #mabg-mdect, rat monoclonal); RRID:AB_2753143.
- CX3CR1-APC (R&D, FAB5825A, rabbit polyclonal, Lot#ACNQ0313081); RRID:AB_2810937.
- GALECTIN-3 (R&D, AF1197, goat polyclonal, Lot#JAA0116021); RRID:AB_ 2234687.
- GFP (Abcam, ab6673, goat polyclonal, Lot#GR287379-16); RRID:AB_305643.
- IBA1 (Abcam, ab5076, goat polyclonal, Lot#GR230719-2); RRID:AB_2224402.
- IBA1 (Wako, 01919741, rabbit polyclonal, Lot#WDE1198); RRID:AB_839504.
- p75NTR (Promega, G3231, rabbit polyclonal, Lot#0000086012); RRID:AB_430853.

- Alexa Flour® 488 (A-11055, Thermo Fisher Scientific, donkey anti-goat polyclonal); RIDD:AB_2534102.
- Alexa Flour® 488 (A-21206, Thermo Fisher Scientific, donkey anti-rabbit polyclonal); RIDD:AB_2535792.
- Alexa Flour® 555 (A-31572, Thermo Fisher Scientific, donkey anti-rabbit polyclonal); RIDD:AB_162543.
- Alexa Flour® 568 (A-11057, Thermo Fisher Scientific, donkey anti-goat polyclonal); RIDD:AB_2534104.
- Alexa Flour® 594 (A-21207, Thermo Fisher Scientific, donkey anti-rabbit polyclonal); RIDD:AB_141637.
- Alexa Flour® 647 (A-21447, Thermo Fisher Scientific, donkey anti-goat polyclonal); RIDD:AB_2535864.

- Secondary antibody conjugated to biotin (Vector laboratories, BA-9200, goat-anti mouse); RRID:AB_2336171.
- Secondary antibody conjugated to biotin (Jackson ImmunoResearch Laboratories, 705-065-003, donkey anti-goat polyclonal); RRID: AB_2340396.

Validation

All antibodies used were validated for use in histological analysis or FACS with mouse samples, which are shown on the website provided by respective companies. Furthermore, we provide number of citations as curated from CiteAb (https://www.citeab.com/), Promega (for p75NTR; G3231) and ThermoFisher Scientific (for CD68; 14-0681-82). In addition:

- ARG1 (ab91279, RRID:AB_10674215): 20 citations. This antibody has been validated for this study in IF in Arg1flox/flox;CX3CR1CreER+/– animals, as well as in other applications by others (e.g. WB, PMID: 24224027).
- ARG1 (sc-18354, RRID:AB_2227469): 12 citations. This antibody has been validated for this study in IF in Arg1flox/flox;CX3CR1CreER +/– animals, as well as in other applications by others (e.g. WB, PMID: 17015747).
- ARG1 (sc-271430, RRID:AB_10648473): 25 citations. It has been validated by WB (e.g. PMID: 30429607).
- CD206 (AF2535, RRID:AB_2063012): 47 citations.
- CD206-BV421 (141717, RRID:AB_2562232): 4 citations. This antibody was validated post-hoc in-house by qPCR and RNAseq.
- CD68 (14-0681-82, RIDD:AB_2572857): 9 references.
- ChAT (MAB5350, RRID:AB_95218): 5 citations.
- CLEC7A (mabg-mdect, RRID:AB_2753143): 14 citations.
- CX3CR1-APC (FAB5825A, RRID:AB_2810937): 3 citations. This antibody was validated post-hoc in-house by qPCR and RNAseq.
- GALECTIN-3 (AF1197, RRID:AB_2234687): 15 citations. This antibody has been validated in-house in GAL-3 knockout animals.
- GFP (ab6673, RRID:AB_305643): 241 citations.
- IBA1 (ab5076, RRID:AB_2224402): widely used marker for myeloid cells with over 300 citations for immunofluorescence for this reagent in various species including mice.
- IBA1 (01919741, RRID:AB_839504): widely used marker for myeloid cells with over 300 citations for immunofluorescence for this reagent in various species including mice.
- p75NTR (G3231, RRID:AB_430853): 15 citations.

Eukaryotic cell lines

Policy information about cell lines and Sex and Gender in Research

Cell line source(s)

State the source of each cell line used and the sex of all primary cell lines and cells derived from human participants or vertebrate models.

Authentication	*Describe the authentication procedures for each cell line used OR declare that none of the cell lines used were authenticated.*
Mycoplasma contamination	*Confirm that all cell lines tested negative for mycoplasma contamination OR describe the results of the testing for mycoplasma contamination OR declare that the cell lines were not tested for mycoplasma contamination.*
Commonly misidentified lines (See ICLAC register)	*Name any commonly misidentified cell lines used in the study and provide a rationale for their use.*

Palaeontology and Archaeology

Specimen provenance	*Provide provenance information for specimens and describe permits that were obtained for the work (including the name of the issuing authority, the date of issue, and any identifying information). Permits should encompass collection and, where applicable, export.*
Specimen deposition	*Indicate where the specimens have been deposited to permit free access by other researchers.*
Dating methods	*If new dates are provided, describe how they were obtained (e.g. collection, storage, sample pretreatment and measurement), where they were obtained (i.e. lab name), the calibration program and the protocol for quality assurance OR state that no new dates are provided.*

☐ Tick this box to confirm that the raw and calibrated dates are available in the paper or in Supplementary Information.

Ethics oversight	*Identify the organization(s) that approved or provided guidance on the study protocol, OR state that no ethical approval or guidance was required and explain why not.*

Note that full information on the approval of the study protocol must also be provided in the manuscript.

Animals and other research organisms

Policy information about studies involving animals; ARRIVE guidelines recommended for reporting animal research, and Sex and Gender in Research

Laboratory animals	Mice were used and maintained under a 12-h light/dark cycle at 22–25 °C with access to food and water ad libitum. Mice used in this study:<br>Mus musculus, C57BL/6J (Charles River), P10, P28, P100 females.<br>Mus musculus, C57BL/6J (Charles River), P10, P28, males.<br>Mus musculus, YARG (The Jackson Laboratory, stock # 015857), P10, P13, males.<br>Mus musculus, YARG (The Jackson Laboratory, stock # 015857), P10, P13, females.<br>Mus musculus, CX3CR1-GFP (The Jackson Laboratory, stock # 005582), P10, males.<br>Mus musculus, Arg1flox/flox;CX3CR1CreER+/−, P10, P20, 2 to 3 month-old, females.<br>Mus musculus, Arg1flox/flox;CX3CR1CreER+/−, P10, P20, 2 to 3 month-old, males.<br>Mus musculus, Arg1flox/flox;CX3CR1CreER-/−, P10, P20, 2 to 3 month-old, females.<br>Mus musculus, Arg1flox/flox;CX3CR1CreER-/−, P10, P20, 2 to 3 month-old, males.
Wild animals	This study did not involve wild animals.
Reporting on sex	*Indicate if findings apply to only one sex; describe whether sex was considered in study design, methods used for assigning sex. Provide data disaggregated for sex where this information has been collected in the source data as appropriate; provide overall numbers in this Reporting Summary. Please state if this information has not been collected. Report sex-based analyses where performed, justify reasons for lack of sex-based analysis.*
Field-collected samples	This study did not involve samples collected form the field.
Ethics oversight	All animal experimental protocols in the present study were in accordance to the respective national, federal and institutional regulations, i.e. the Guidelines of the European Union Council, following Swedish regulations for the use of laboratory animals and approved by the Regional Animal Research Ethical Board, Stockholm, Sweden (Ethical permits N248/13) the Spanish regulations (BOE 34/11370–421, 2013) and in conformity with the Canada Council on Animal Care guidelines.

Note that full information on the approval of the study protocol must also be provided in the manuscript.

Clinical data

Policy information about clinical studies

All manuscripts should comply with the ICMJE guidelines for publication of clinical research and a completed CONSORT checklist must be included with all submissions.

Clinical trial registration	*Provide the trial registration number from ClinicalTrials.gov or an equivalent agency.*
Study protocol	*Note where the full trial protocol can be accessed OR if not available, explain why.*
Data collection	*Describe the settings and locales of data collection, noting the time periods of recruitment and data collection.*

Outcomes | *Describe how you pre-defined primary and secondary outcome measures and how you assessed these measures.*

Dual use research of concern

Policy information about dual use research of concern

Hazards

Could the accidental, deliberate or reckless misuse of agents or technologies generated in the work, or the application of information presented in the manuscript, pose a threat to:

No	Yes	
☐	☐	Public health
☐	☐	National security
☐	☐	Crops and/or livestock
☐	☐	Ecosystems
☐	☐	Any other significant area

Experiments of concern

Does the work involve any of these experiments of concern:

No	Yes	
☐	☐	Demonstrate how to render a vaccine ineffective
☐	☐	Confer resistance to therapeutically useful antibiotics or antiviral agents
☐	☐	Enhance the virulence of a pathogen or render a nonpathogen virulent
☐	☐	Increase transmissibility of a pathogen
☐	☐	Alter the host range of a pathogen
☐	☐	Enable evasion of diagnostic/detection modalities
☐	☐	Enable the weaponization of a biological agent or toxin
☐	☐	Any other potentially harmful combination of experiments and agents

ChIP-seq

Data deposition

☐ Confirm that both raw and final processed data have been deposited in a public database such as GEO.

☐ Confirm that you have deposited or provided access to graph files (e.g. BED files) for the called peaks.

Data access links<br>*May remain private before publication.*	*For "Initial submission" or "Revised version" documents, provide reviewer access links. For your "Final submission" document, provide a link to the deposited data.*
Files in database submission	*Provide a list of all files available in the database submission.*
Genome browser session<br>(e.g. UCSC)	*Provide a link to an anonymized genome browser session for "Initial submission" and "Revised version" documents only, to enable peer review. Write "no longer applicable" for "Final submission" documents.*

Methodology

Replicates	*Describe the experimental replicates, specifying number, type and replicate agreement.*
Sequencing depth	*Describe the sequencing depth for each experiment, providing the total number of reads, uniquely mapped reads, length of reads and whether they were paired- or single-end.*
Antibodies	*Describe the antibodies used for the ChIP-seq experiments; as applicable, provide supplier name, catalog number, clone name, and lot number.*
Peak calling parameters	*Specify the command line program and parameters used for read mapping and peak calling, including the ChIP, control and index files used.*
Data quality	*Describe the methods used to ensure data quality in full detail, including how many peaks are at FDR 5% and above 5-fold enrichment.*
Software	*Describe the software used to collect and analyze the ChIP-seq data. For custom code that has been deposited into a community repository, provide accession details.*

Flow Cytometry

Plots

Confirm that:

☒ The axis labels state the marker and fluorochrome used (e.g. CD4-FITC).

☒ The axis scales are clearly visible. Include numbers along axes only for bottom left plot of group (a 'group' is an analysis of identical markers).

☒ All plots are contour plots with outliers or pseudocolor plots.

☐ A numerical value for number of cells or percentage (with statistics) is provided.

Methodology

Sample preparation	For RNA-Seq and RT-qPCR, brains were pooled and roughly minced with a scalpel, followed by mechanical dissociation with use of a tissue grinder. The tissue was further homogenized by pipette trituration and passed through cell strainer. Whole-brain homogenate was separated by 20 % Percoll (Percoll PLUS, low endotoxin) gradient centrifugation at 500 g for 20 min at 4 °C (no brake). The pellet was washed and resuspended in cold FACS staining buffer (R&D Systems). Cells were stained with primary antibodies against CX3CR1 (R&D, FAB5825A) and CD206 (Biolegend, 141717) for 45 min at 4 °C.
Instrument	FACSAria III Cell Sorter system.
Software	FACSDiva™ software (BD Biosciences) was used to collect and analyze the data.
Cell population abundance	Post-hoc RNA-Seq data analysis and qPCR confirmed the purity of the samples sorted.
Gating strategy	Gating strategy for this study is shown in Extended Data Fig. 5a. CNS cells were gated (singlets), followed by being gated for CD206 (negative selection), CX3CR1 (positive selection) prior to be divided into ARG1-YFP-positive and ARG1-YFP-negative.

☒ Tick this box to confirm that a figure exemplifying the gating strategy is provided in the Supplementary Information.

Magnetic resonance imaging

Experimental design

Design type	*Indicate task or resting state; event-related or block design.*
Design specifications	*Specify the number of blocks, trials or experimental units per session and/or subject, and specify the length of each trial or block (if trials are blocked) and interval between trials.*
Behavioral performance measures	*State number and/or type of variables recorded (e.g. correct button press, response time) and what statistics were used to establish that the subjects were performing the task as expected (e.g. mean, range, and/or standard deviation across subjects).*

Acquisition

Imaging type(s)	*Specify: functional, structural, diffusion, perfusion.*
Field strength	*Specify in Tesla*
Sequence & imaging parameters	*Specify the pulse sequence type (gradient echo, spin echo, etc.), imaging type (EPI, spiral, etc.), field of view, matrix size, slice thickness, orientation and TE/TR/flip angle.*
Area of acquisition	*State whether a whole brain scan was used OR define the area of acquisition, describing how the region was determined.*

Diffusion MRI ☐ Used ☐ Not used

Preprocessing

Preprocessing software	*Provide detail on software version and revision number and on specific parameters (model/functions, brain extraction, segmentation, smoothing kernel size, etc.).*
Normalization	*If data were normalized/standardized, describe the approach(es): specify linear or non-linear and define image types used for transformation OR indicate that data were not normalized and explain rationale for lack of normalization.*
Normalization template	*Describe the template used for normalization/transformation, specifying subject space or group standardized space (e.g. original Talairach, MNI305, ICBM152) OR indicate that the data were not normalized.*
Noise and artifact removal	*Describe your procedure(s) for artifact and structured noise removal, specifying motion parameters, tissue signals and*

Noise and artifact removal

physiological signals (heart rate, respiration).

Volume censoring

Define your software and/or method and criteria for volume censoring, and state the extent of such censoring.

Statistical modeling & inference

Model type and settings

Specify type (mass univariate, multivariate, RSA, predictive, etc.) and describe essential details of the model at the first and second levels (e.g. fixed, random or mixed effects; drift or auto-correlation).

Effect(s) tested

Define precise effect in terms of the task or stimulus conditions instead of psychological concepts and indicate whether ANOVA or factorial designs were used.

Specify type of analysis: ☐ Whole brain ☐ ROI-based ☐ Both

Statistic type for inference
(See Eklund et al. 2016)

Specify voxel-wise or cluster-wise and report all relevant parameters for cluster-wise methods.

Correction

Describe the type of correction and how it is obtained for multiple comparisons (e.g. FWE, FDR, permutation or Monte Carlo).

Models & analysis

n/a	Involved in the study
☐	☐ Functional and/or effective connectivity
☐	☐ Graph analysis
☐	☐ Multivariate modeling or predictive analysis

Functional and/or effective connectivity

Report the measures of dependence used and the model details (e.g. Pearson correlation, partial correlation, mutual information).

Graph analysis

Report the dependent variable and connectivity measure, specifying weighted graph or binarized graph, subject- or group-level, and the global and/or node summaries used (e.g. clustering coefficient, efficiency, etc.).

Multivariate modeling and predictive analysis

Specify independent variables, features extraction and dimension reduction, model, training and evaluation metrics.
